# Intelligent vulnerability assessment system for college students mental health based on few-sample reinforcement learning approach

**DOI:** 10.3389/fpsyt.2026.1932839

**Published:** 2026-09-25

**Authors:** Xiaobo Huang, Jiayue Ji, Shi Cheng

**Affiliations:** 1 Student Management Section, Office of Student Affairs, Nantong University, Nantong, China; 2 School of Artificial Intelligence and Computer Science, Nantong University, Nantong, China

**Keywords:** college student mental health, few-shot learning, meta-learning, ordinal classification, psychological vulnerability assessment, reinforcement learning, sequential decision-making

## Abstract

**Introduction:**

College students’ psychological vulnerability assessment is constrained by data scarcity, high labeling cost, privacy sensitivity, and small, imbalanced samples, and existing frameworks typically treat vulnerability as a static judgment that cannot track how a student’s condition changes over time. This study examined whether a few-sample hierarchical grading model combined with meta-reinforcement-learning-based sequential decision-making could address both problems jointly.

**Methods:**

A few-sample hierarchical grading model, combining meta-learning and transfer regularization with a prototype-based embedding and ordinal threshold assignment, quantified static risk from a handful of labeled examples per grade, while a sequential risk-identification method formulated dynamic assessment as a Markov decision process solved by a two-stage meta-reinforcement-learning policy. Case studies used a longitudinal, class-imbalanced cohort simulation calibrated to the documented statistics of a mobile-sensing mental-health study (no real participant data were used) and compared the proposed models against threshold-rule, supervised, fine-tuned, few-shot, and non-meta reinforcement-learning baselines, with sensitivity analyses on support-set size, reward weights, discount factor, and regularization weight, plus a regime-switching robustness check.

**Results:**

The proposed grading model attained a macro-F1 of 0.847 with ten labeled examples per grade, over twenty percentage points above a conventional deep-learning classifier, though its edge over other episodic few-shot architectures was small and inconsistent, indicating episodic meta-learning as the main source of this gain. The meta-reinforcement-learning policy achieved a higher alert precision-recall area (0.514 vs. 0.380) than a non-meta baseline and avoided its degenerate always-escalate behavior, but neither the main study nor the regime-switching check showed the composite reward lengthening lead time versus a simpler accuracy-only reward, which matched its lead time at a much lower escalation rate. Sensitivity analyses showed the reward-weight ratio and discount factor shaping the early-identification versus intervention-cost trade-off.

**Conclusion:**

These findings suggest that combining few-sample meta-learning with metareinforcement-learning-based sequential decision-making can improve vulnerability assessment under scarce, evolving data relative to conventional static and non-meta-adapted approaches, with an accuracy-only reward recommended as the practical default. The case studies relied on a calibrated simulation rather than raw longitudinal records, so prospective validation on real campus data under appropriate privacy safeguards is needed before responsible deployment.

## 1 Introduction

Nowadays, mental health problem of college students has become a more and more urgent global concern, and elevated rates of depressive symptoms, anxiety, and suicidal ideation have been reported across diverse student populations, which are also shown to cluster with broader health risk behaviors (1). Besides, public health disruptions, such as the prolonged campus quarantines during the COVID-19 pandemic, have further increased the psychological strain on students and exposed the fragility of existing campus support mechanism (2). Not only individual well-being is affected, unresolved psychological vulnerability also carries a real institutional consequence: administrative evidence has linked persistent student mental health difficulties with elevated dropout from higher education (3), and delayed identification will bring a real cost to the institution. Because of this, universities and policymakers have gradually moved from *ad hoc* counseling arrangement toward a more formalized, institution-wide mental health policy that is aimed at systematizing the early identification and intervention (4). In recent years, this policy shift has also started to meet with technological innovation, since artificial-intelligence-enabled tools are more and more explored as a way to extend the reach and responsiveness of campus mental health support (5). Under this background, it becomes a pressing and practical need for higher education institutions to develop an assessment approach that can identify the psychologically vulnerable students in an accurate, efficient, and large-scale way.

Standardized psychological scales and questionnaires are still the most used instrument by which universities carry out large-scale mental health screening, and tools such as the General Health Questionnaire are routinely used to classify students by their risk level (6). However, more and more psychometric studies have found that this kind of instrument cannot always generalize well across different subpopulations, since reliability and measurement invariance analyses in college student athletes have shown that questionnaire batteries which are validated on general population may not hold consistently across different student groups (7). Besides, symptom-network analyses of large student cohorts have further argued that the total-score paradigm which most scales are based on will obscure the specific symptoms that most strongly drive an individual’s overall risk, so the diagnostic precision of aggregate scores becomes limited (8). What makes this problem worse is that evidence from universal self-report screening programs shows that scale-based assessment can also bring some unintended harm, including false positives and screening-related distress, when it is applied to entire student populations without careful consideration (9). These limitations have made researchers try to shorten and personalize the assessment scales in order to reduce the burden on respondents while the diagnostic value is preserved, but such adaptation still relies on the same static, single-timepoint measurement logic, and it does little to solve the underlying cost of large-scale data collection and expert labeling (10).

In order to go beyond the limitation of self-report instrument, many works have already applied machine learning technique to predict the psychological distress and risk directly from survey, cohort, or longitudinal data, and it is shown that data-driven models can capture the risk patterns which the static scale score alone usually cannot catch (11, 12). Electronic-health-record-based models have also extended this line of work into clinical setting, showing that mental health crisis can be anticipated in advance from the patient data that is routinely collected, instead of relying only on manual chart review (13), and at the same time, cross-cultural machine learning studies have tried to find out which risk factor can most reliably predict the adolescent mental health outcome across different population (14). Specifically in higher education, deep learning approaches have been proposed for detecting the stress severity from physiological pulse signal and for automatically identifying depressive symptom from student’s online behavioral trace, so that the assessment can move away from self-assessment scale and toward the continuously collected behavioral evidence (15, 16). Multimodal deep learning framework which integrates biological, lifestyle, text, speech, and physiological signal has also pushed the prediction performance further, and this shows the potential of combining heterogeneous data stream for early mental health risk detection (17, 18). Even so, a recent and comprehensive review of this literature concludes that the reported gain of machine learning and deep learning model is, in almost every case, depending on the access to large and well-labeled training dataset, and this assumption is hard to satisfy in campus mental health context, because data collection there is constrained by privacy sensitivity, stigma, and limited institutional resource (19).

This dependency on data has made researchers turn to few-shot learning, meta-learning, and other related low-resource paradigm, which are designed on purpose to reduce the reliance on large annotated corpus. Lightweight, instruction-tuned language model and prompt-based learning framework have been proposed to detect depression from limited labeled data, and it is reported that they can achieve competitive accuracy while the computational and annotation cost is reduced a lot (20, 21). Based on this direction, few-shot and zero-shot prompting strategy have also been applied to identify depression and anxiety symptom in patient communication and to distinguish depression subtype from social media text, and several studies have compared few-shot prompting with conventional fine-tuning directly, reporting that a carefully designed prompt can match or even approach the fine-tuned performance under scarce-label condition (22, 23). Related work has used large-language-model-based data augmentation to relieve the scarcity of labeled suicidal ideation text, and multitask learning over pretrained audio and text embedding has been used to predict depression severity and suicide risk together from small clinical sample (24, 25). Not only for text application, few-shot learning has also been proven effective for emotion recognition from spontaneous speech and for decoding the mental state from fMRI signal under limited-data condition, and a broader survey of few-shot learning for biomedical time series confirms that this paradigm is more and more viewed as a possible way to build clinically meaningful model when large labeled dataset is not available (26–28). Even so, almost all of these efforts still treat the psychological assessment as a one-shot, static classification problem, which means predicting one single risk label from a fixed input, and they do not go further to model how an individual’s psychological vulnerability changes and needs to be tracked as time goes on.

Reinforcement learning can offer a complementary paradigm here, because it is designed for sequential decision-making instead of single-shot prediction, and its application on health-related risk assessment has started to get attention recently. A systematic review of reinforcement learning in critical care has concluded that this technique is very suitable for the problem which needs repeated, state-dependent decision, although the review also points out that the clinical adoption is still at an early stage (29). Disease progression has already been modeled as a sequential reasoning process over a medical knowledge graph, in which an agent takes a series of interpretable step to arrive at the risk prediction, and this shows how sequential decision formulation can support dynamic, explainable risk trajectory rather than a static label (30). At the same time, adaptive trial design which adjusts the intervention strategy step by step based on the accumulating evidence, and Bayesian sequential monitoring scheme which keeps updating risk estimate as new surveillance data arrives, both demonstrate that it is valuable to treat the health-related risk identification as an evolving process instead of a one-time assessment (31, 32). Outside the health domain, reinforcement learning has also been used for identifying and predicting the dynamically evolving risk in cost-sensitive credit decision and in corporate low-carbon transition management, and both of them model the risk identification explicitly as a sequential decision problem, with an agent that keeps updating its assessment policy through interacting with the incoming data (33, 34). However, application of reinforcement learning specifically on psychological vulnerability assessment is still very scarce, and none of the studies reviewed above has combined the sequential decision-making with the low-resource learning capability that is needed to work under the data constraint of college mental health setting.

If we put all the reviewed literature together, three limitations can be found that current approach to college student mental health assessment has not solved yet. First, traditional scale- and questionnaire-based assessment is still constrained by difficult data acquisition, high labeling cost, privacy sensitivity, and small and imbalanced sample, and it also suffers from the reliability gap across different subpopulation and the risk of screening-related harm. Second, although machine learning and deep learning model can perform better than static scale, their reported gain depends heavily on large labeled dataset that is rarely available in campus mental health context, and even the newly emerging few-shot and meta-learning solution is mostly limited to one-shot, static classification, without being extended systematically to psychological vulnerability grading under real data scarcity. Third, almost all existing evaluation framework, no matter it is scale-based or data-driven, treats the psychological assessment as a single-point-in-time judgment, so the dynamic and individually different evolution of psychological vulnerability is left largely unaddressed, even though reinforcement-learning-based sequential decision-making has already shown its promise for dynamic risk identification in other health and non-health domain.

In order to address these gaps, this paper proposes an intelligent vulnerability assessment system for college students’ mental health, which integrates few-shot learning with reinforcement-learning-based sequential decision-making. The main contribution of this paper can be summarized as follows: 1) a few-shot learning-based hierarchical assessment model is established for grading the college students’ psychological vulnerability, in which episodic meta-learning is the primary source of the model’s fewsample generalization advantage over a conventional, non-episodically-trained classifier, and transfer regularization anchored to a pretrained backbone is incorporated as a complementary refinement that is concentrated at the smallest support sizes, so the reliance on large-scale labeled data can be alleviated and the model’s generalization ability under few-shot condition can also be improved; 2) based on this hierarchical assessment model, a reinforcement-learning-based method for temporal identification of psychological risk is further proposed, which formulates the identification process as a sequential decision problem with the design of state, action, and reward, so that an agent can interact with the assessment environment and adjust its strategy dynamically, in order to track and predict the evolving trajectory of a student’s potential psychological risk; the empirical role of the reward’s cost and timeliness terms relative to an accuracy-only formulation, and the practical operating point this implies, are examined in detail in Section 5 and are found to favor a more conservative reward configuration than the full composite reward for most deployment settings.

The rest of this paper is organized in the following way. Section 2 describes the problem background and formalizes the college student mental health vulnerability assessment problem, and it characterizes both the static grading task and the dynamic risk identification task that together motivate the proposed modeling framework. Section 3 presents the mathematical formulation of the few-shot hierarchical assessment model and the reinforcement-learning-based sequential risk identification method, together with the corresponding solution approach. Section 4 reports the simulation-based case studies which validate the effectiveness of the proposed system, and it also conducts sensitivity analysis with respect to the key parameters and sample conditions, reporting the resulting metric primarily in descriptive terms. Section 5 then interprets these results in relation to the operational trade-offs a deploying institution would face, the few-shot and sequential clinical-decision-making literature reviewed above, and the limitations of a simulation-based validation. Finally, Section 6 concludes the paper by summarizing the main finding and discussing the direction for future research.

## 2 Problem description

### 2.1 Multi-source data-driven formulation of the psychological vulnerability assessment problem

The psychological vulnerability assessment problem addressed in this paper comes from a concrete institutional need: a university counseling office must decide, for each student who is enrolled, how psychologically at risk that student currently is, by using whatever information the institution can realistically collect. In practice, this information is neither abundant nor uniform. There are three broad categories of data that are typically available. The first category is the historical psychological scale record, which means the score or item-level response from the periodic mental health screening that a subset of students have completed at some point during their enrollment. The second category is the campus behavioral log, including the library and dormitory access pattern, academic performance trajectory, campus card consumption regularity, and online learning platform engagement, and all of them are passively generated as a by-product of ordinary student life. The third category is the peer- and counselor-reported qualitative note, such as the referral record from resident advisor or the brief case note from voluntary counseling session. The modeling framework in this section and in Section 3 is defined over all three sources; the case studies in Section 4, however, are conducted on a calibrated simulation environment that reproduces only the first two sources, because the publicly documented dataset used for calibration does not release peer- or counselor-reported qualitative note, and this scope restriction is stated explicitly where the case-study data are described (Section 4.1). None of these three sources is complete for the whole student body, none of them is collected on a fixed schedule, and only a small part of students have a verified psychological risk label at any given time, because obtaining such a label usually needs a clinical interview or a validated scale given by trained staff. So the resulting dataset is small in absolute size, sparsely and unevenly labeled among students, and skewed toward the majority of students who are not in visible distress, and this combination of property is exactly what makes conventional large-sample supervised learning hard to apply directly on this task.

Besides the general problem of scarcity, each of the three data sources also brings its own specific obstacle. Historical scale record is the most directly interpretable one in psychological term, but it is only collected once or twice per academic year during the scheduled screening event, so the interval between two consecutive scale administration for the same student can be many months, and during this period the student’s condition may already change a lot without being recorded. Behavioral log, on the other hand, is continuously generated and therefore much more abundant in volume, but it is only a weak and indirect proxy of psychological state: a decline of library visit or dormitory check-in could reflect an emerging withdrawal and distress, but it could also just reflect an unrelated change of study habit, a part-time job, or simply a personal preference, and no behavioral signal alone can carry a verified label to tell which interpretation is correct. Peer- and counselor-reported note carries the richest qualitative signal about a student’s real condition, but it is also the scarcest one among the three sources, since it depends on a referral or a voluntary counseling visit that has already happened, and it is recorded in free-form language which must be coded into a usable feature before it can be combined with the other two sources. So the general problem formulation introduced next must not only handle a small total sample size, but also this kind of asymmetry in reliability, frequency, and interpretability among the three sources that together make up the feature vector *x*.

To think about this situation in a way that does not depend on any particular modeling choice, it is useful to first write down the assessment task in its most general form. Let each student be characterized, at the moment of assessment, by a feature representation *x* which is obtained by putting the three data sources described above together into one single vector, and let *y* denote the psychological risk label that the assessment system is supposed to infer for that student. Then the general assessment problem can be stated as constructing a mapping from *x* to *y* that is accurate for the students whose true label is not known, by only using the limited set of students for whom a label happens to be available. This formulation leaves the internal structure of the mapping unspecified on purpose, because the purpose of Section 2 is only to characterize the problem before we commit to a model, and the internal structure will be given only in Section 3. What matters at this stage is that the mapping has to be learned from an evaluation set which is small compared with the diversity of the student population, and also that the feature representation *x* itself is heterogeneous, combining the numerical scale score, count-based behavioral statistic, and coarsely coded qualitative indicator all within the same vector.

A large-capacity model that is naively applied cannot solve this situation, because the temptation to simply “collect more data and train a bigger network” is the default response in many other application domains. A high-capacity classifier that is trained end-to-end on the pooled labeled subset described above only has, in effect, as many independent training signals as there are labeled students, and in a typical campus counseling office this may be just a few hundred case spread unevenly across risk grade and academic cohort. Under this condition, a flexible model tends to memorize the idiosyncratic combination of behavioral and scale feature that is associated with the small number of observed high-risk case, instead of learning a decision boundary that can generalize to the next cohort of student, whose behavioral pattern, course load, and life circumstance are different from those in the training set. This kind of failure is exactly why the problem should be treated as a family of related few-sample task, rather than one large classification problem that is solved once and reused forever, and this is also the reason why the general formulation introduced next is left open on purpose about how the mapping from *x* to *y* is learned.

Both the objective and the constraint of this general problem come from the practical circumstance of campus mental health work, not from any convenience of modeling. The objective is to keep the assessment accurate and, what is more important, to generalize well to the students whose data pattern has never been seen before, even though the number of labeled example that is available for training is small. This objective cannot be given up, because a counseling office cannot wait for assessment until a large labeled dataset has been accumulated, and the students who are currently at risk need to be identified with whatever data already exist now. The constraint, on the other hand, comes from three sources that were already mentioned in Section 1 and are now translated into property of the problem: the data privacy consideration limits how much additional psychological data can be collected beyond what is already routinely gathered, which caps the sample size that can be achieved; the practical need of timely intervention limits how long the system is allowed to wait to accumulate more labeled case before it produces a usable assessment; and the genuine individual difference means the same behavioral pattern, for example a period of reduced social activity, can correspond to very different risk level for different student, so a single global mapping which is estimated from too few example per subpopulation may become systematically miscalibrated for exactly the student it is supposed to protect. **Figure 1** summarizes this general formulation as a three-stage pipeline, which connects the data layer, a feature aggregation stage that produces the unified representation *x*, and an assessment mapping stage that produces the output *y*, and the constraint of data scarcity, labeling cost, and class imbalance are explicitly marked as external pressure acting on this pipeline, instead of being internal modeling choice.

**Figure 1:**
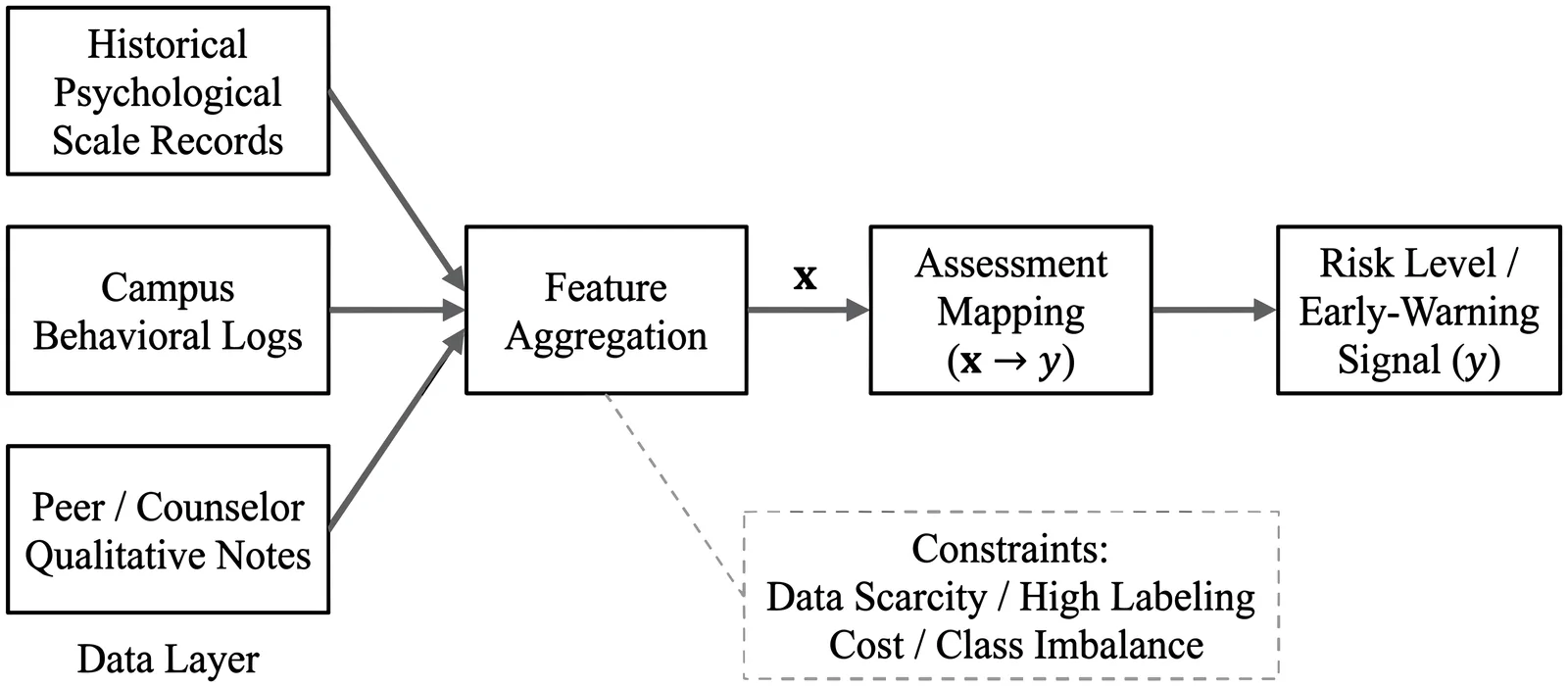
Overall architecture of the data-driven college student psychological vulnerability assessment problem.

The general framework shown in **Figure 1** is on purpose left open about how the assessment mapping is learned and about whether the resulting assessment refers to one single point in time or to a condition that is evolving. These two open questions are exactly where the present research is different from a conventional supervised-learning treatment of the problem, and both of them are also the entry point for the two innovations introduced in this paper. If left unresolved, the general framework in **Figure 1** would just become an ordinary classifier trained on whatever labeled data is available, which, as it is argued in Section 1, tends to overfit under the sample size that is realistically available in campus setting, and it can only produce a static, one-shot judgment which cannot track how a student’s condition changes after the first assessment. So Section 2.2 will now reformulate these two open questions explicitly, and each of them is translated into a change of the structure of the general problem, instead of being left as only a narrative motivation.

### 2.2 Problem reformulation for few-sample hierarchical grading and sequential risk identification

The first reformulation is about how the mapping from *x* to *y* can be learned when the labeled example is scarce. Instead of treating the assessment problem as one single classification task trained on one pooled dataset, this paper reformulates it as a distribution of related few-sample task. Each task is built by drawing, from the bigger pool of partially labeled student record, a small support set of labeled example together with a query set of example whose label needs to be inferred, so a task with *C* risk grade and *K* labeled example per grade is called a *K*-shot, *C*-way task. Framing the problem in this way changes what is actually being learned: instead of estimating one fixed decision boundary from one small labeled set, the system is now asked to learn how to construct a decision boundary quickly from whatever small labeled set it is given, by practicing this construction again and again across many such task sampled from a task distribution. This reformulation makes it possible to apply meta-learning and transfer learning to the problem in a meaningful way, since both technique are based on this task-distribution view rather than one single fixed training set, and this is also the structural embodiment of the first innovation claimed in Section 1.

In a practical way, the task $T_{n}$ described above does not need to be constructed artificially, because it can arise naturally from how campus mental health data already accumulates. Each academic term, each college inside the university, or each counseling cohort that goes through a screening cycle can be treated as producing one task, since the students within this kind of group share a common assessment period and a common pool of partially labeled record from which a support set and a query set can be drawn. Framing the historical data in this way means that the episodic task structure needed for meta-learning is obtained basically for free from the institution’s already existing organizational unit, instead of needing an artificial re-partitioning of the data that has no correspondence with how the university actually operates, and this observation is what makes the few-sample reformulation practically implementable, not just a theoretical convenience.

A closely related, but different, structural change is about the nature of the label space itself. The general formulation in Section 2.1 left *y* unspecified about whether it is a nominal category or an ordered grade. In this paper, *y* is set explicitly to be an ordinal risk grade, for example ranging from low to severe vulnerability, so misclassifying a severely at-risk student as only low-risk is a qualitatively more serious mistake than misclassifying a moderate case as low, even though both of them are technically a classification error of one grade. To represent this ordering is a structural requirement, not just a modeling detail, because a plain multi-class classifier trained under few-sample condition has no mechanism to prefer the “close” error over the “far” error, and under scarce label the two kinds of error can be easily confused. So the reformulated problem requires that the decision function respects the ordering of the grade set, and that adjacent grade are separated by a graded decision structure instead of an arbitrary one; this requirement will be carried into the ordinal thresholding construction of Section 3.1, but it is already introduced here as a defining property of the assessment task, which makes it different from an unordered few-shot classification problem.

A simpler alternative for the ordinal reformulation, that is, training several independent binary classifier for each pair of adjacent grade and combining their output, is not chosen here for a related reason. Under the sample size that is available for each grade in a campus setting, splitting the already limited support set into several separate binary comparisons would leave each individual classifier with even fewer effective example than the joint prototype-based representation described above, and this makes the data-scarcity constraint worse instead of better, which is exactly what motivated the few-sample reformulation at the beginning. Keeping one single shared embedding space where all grade are represented at the same time, and only imposing the ordinal structure at the final assignment step, allows every labeled example in the support set to help shape this same representation no matter which grade it belongs to, and this is a more sample-efficient use of the scarce labeled data than training a separate classifier for every grade pair.

The second reformulation is about the temporal nature of psychological vulnerability. The general mapping from *x* to *y* in Section 2.1 implicitly treats the assessment as one single, self-contained event: a feature vector goes in, a risk grade comes out, and the problem is finished there. This kind of treatment cannot represent the phenomenon that motivated this research at the very beginning, that is, a student’s psychological condition evolves, sometimes gradually and sometimes suddenly, and the value of an assessment system partly lies in noticing this evolution before it turns into a crisis. So this paper reformulates the assessment problem, at the level of one individual student who is observed repeatedly over an academic term, as a sequential decision problem which unfolds over discrete assessment cycle indexed by *t*. At each cycle, an agent representing the assessment system observes a state that summarizes what is currently known about the student, chooses an action which represents an assessment decision such as keeping the current risk judgment, escalating it, or asking for additional observation, and then receives a reward that reflects how well this decision serves the two goals of accuracy and timeliness together. Framing the risk identification in this way replaces the single-shot question “what is this student’s risk level” with the repeated question “given everything observed so far, what is the best assessment decision right now,” and this is the structural basis for applying reinforcement learning to the problem, as well as the embodiment of the second innovation claimed in Section 1.

A natural alternative for the sequential decision formulation would be simply repeating the static grading procedure of Section 3.1 (items 1–3) at every new assessment cycle, and treating the consecutive grades as an ordinary time series to be smoothed or extrapolated. This alternative is not chosen, because it treats every assessment cycle as an isolated event and it does not provide any principled mechanism for weighing the cost of an assessment action, such as the disruption caused by escalating a case or the resource cost of asking for additional observation, against the benefit that the information brings, and it also does not offer a natural way to reward the system for detecting an emerging risk trajectory before it turns into a crisis, instead of only reporting the current grade after it already happened. Treating the temporal problem as a sequential decision process with an explicit reward structure allows both of these considerations to be represented directly inside the model, instead of being handled as an *ad hoc* post-processing step applied on a sequence of otherwise independent static prediction, and it is exactly this property that Section 3.1 (items 4–7) makes use of when constructing the composite reward function.

These two reformulations are not independent from each other; they are coupled by design, and this coupling is itself also part of the contribution of this paper. The few-sample hierarchical grading task produces, for a given student at a given point of time, both a discrete risk grade and a continuous embedding that locates the student inside the learned risk representation space. Instead of throwing away this information once a static grade has been produced, the reformulated sequential decision problem treats the static grade and its underlying embedding as part of the state that is observed by the reinforcement learning agent at the first assessment cycle, so the sequential module does not need to relearn the risk representation from raw behavioral data alone, but can instead build on the representation that is already learned under the few-sample paradigm. **Figure 2** shows this coupling directly: the left panel depicts the few-sample task sampling structure that underlies the first reformulation, with several tasks each containing a small support set and query set of labeled example; the right panel depicts the sequential state-action-reward interaction loop that underlies the second reformulation, unfolding across the successive assessment cycle; and the bold arrow connecting the two panels represents the transfer of the static grade and embedding into the initial state of the sequential process. This coupled structure is exactly what Section 3 will formalize mathematically, first by establishing the few-sample grading model, then by establishing the sequential decision model, and finally by specifying how the two of them are joined together into one single trainable system.

**Figure 2:**
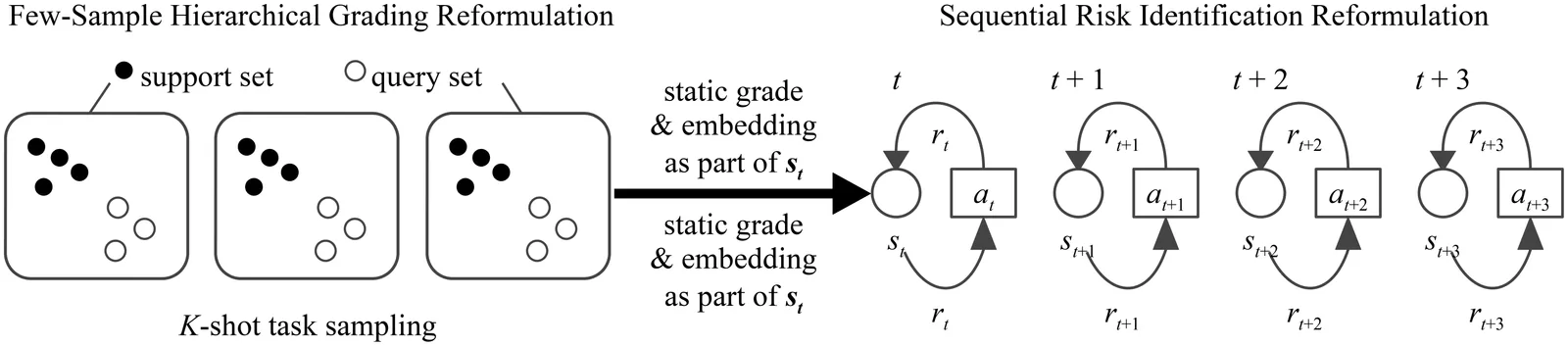
Problem reformulation from few-sample hierarchical grading to sequential risk identification, with the right-hand timeline redrawn under a single consistent relative-subscript scheme, $\left(s_{t},a_{t},r_{t})\;\to \;(s_{t+1},a_{t+1},r_{t+1})\;\to \;(s_{t+2},a_{t+2},r_{t+2})\;\to \;(s_{t+3},a_{t+3},r_{t+3}\right)$, correcting the inconsistent $s_{t},s_{t},s_{2},s_{3}$/ $r_{t},r_{1},r_{2},r_{3}$ labeling flagged by the reviewer.

## 3 Mathematic formulation

### 3.1 Model establishment

#### 3.1.1 Few-sample task structure and embedding representation

Following the reformulation in Section 2.2, the pool of partially labeled student record is organized into a collection of episodic few-sample task, which are drawn from a task distribution *p*(T), and each task T*_n_*is defined by a support set and a query set that are constructed over the same ordinal grade set {1*,…,C*}:

(1) $$S_{n}={\left\{(x_{i}^{\text{s}},y_{i}^{\text{s}})\right\}}_{i=1}^{K\times C},\;Q_{n}={\left\{(x_{j}^{\text{q}},y_{j}^{\text{q}})\right\}}_{j=1}^{M},\;T_{n}=(S_{n},Q_{n})∼p(T)$$

where the superscript s and q denote the membership in the support and query subset respectively, *K* isthe number of labeled example per grade that is available in the support set (also called the “shot” count), *C* is the number of ordinal grade (also called the “way” count), and *M* is the number of query example whose label the model needs to infer within task $T_{n}$. A feature extraction network $f_{\theta }(\cdot )$, which is parameterized by *θ*, maps a raw aggregated feature vector $x\in {\mathbb{R}}^{d}$ into an embedding $z=f_{\theta }(x)\in {\mathbb{R}}^{m}$, and for each grade c∈{1,…,C} inside task $T_{n}$. a class prototype is computed, following the prototype-based metric-learning paradigm for few-sample classification (35), as the mean embedding of the support example that belong to that grade,

(2) $$p_{n,c}=\frac{1}{K}\sum_{(x_{i}^{\text{s}},y_{i}^{\text{s}})\in S_{n},\;y_{i}^{\text{s}}=c}f_{\theta }(x_{i}^{\text{s}}),\;c=1,\ldots ,C$$

so each task is represented, after only *K* labeled example per grade, by a small set of *C* prototype, instead of by a fully re-estimated classifier. Given a query example *x*, its grade is inferred through a distance-based softmax, which gives a higher probability to the grade whose prototype is closer to the query embedding in the learned space,

(3) $$P_{\theta }(y=c|x,S_{n})=\frac{\text{exp}\;\left(-{\left\|f_{\theta }\left(x\right)-p_{n,c}\right\|}_{2}^{2}\right)}{\sum_{c^{\prime }=1}^{C}\text{exp}\;\left(-{\left\|f_{\theta }\left(x\right)-p_{n,c^{\prime }}\right\|}_{2}^{2}\right)},\;c=1,\ldots ,C$$

and this is the mechanism through which the model can achieve few-sample generalization: the classification does not need re-training on new student, but only needs to re-compute the prototype from whichever small labeled set is available at the moment, as long as the embedding network *f_θ_*itself has been trained to produce embedding that clusters meaningfully by grade, even when only a handful of example are seen. The squared Euclidean distance is used in (Equation 3) instead of a more elaborate learned metric, because once *f_θ_*has been trained under the episodic objective of (Equations 4, 5), the burden of capturing the relevant notion of similarity between student is already placed on the embedding network itself, and adding another learned distance function on top of the embedding would bring extra parameters that must also be estimated from the same scarce labeled data, which goes against the sampleefficiency goal that motivates the prototype-based design in the first place. It is also because of this choice that the grading model stays computationally light enough to be recomputed whenever a new support set becomes available, since the prototype computation in (Equation 2) only needs a forward pass through *f_θ_*followed by a class-wise average, without any iterative optimization.

#### 3.1.2 Transfer-regularized meta-training objective

The embedding network *f_θ_*cannot be trained reliably from only the campus-specific labeled data, since this data is exactly the scarce resource that this paper is designed to work around. Instead, it is initialized from a broader pretrained representation *θ*^pre^, which is obtained from related psychological or behavioral prediction task that has more abundant data, and it is then adapted to the campus assessment task through a combination of episodic meta-training and transfer-learning regularization. The source task that produces *θ*^pre^ is defined on the identical *d*-dimensional aggregated feature vector *x* as the target grading task, so no cross-domain feature alignment is required; what differs is only the label the network is trained to predict. Concretely, in the case studies of Section 4, where a calibrated simulation stands in for the inaccessible raw multi-cohort record (Section 4.1), the source task is a regression of the underlying continuous vulnerability trajectory itself, pooled across every meta-training-cohort student-week snapshot (7,259 snapshots drawn from the 109 cohort-A students, capped at the first 70 assessment weeks per student), trained for 200 full-batch Adam epochs at a learning rate of 10^−2^; this exercises the same feature-extraction network on a continuous, more abundantly available auxiliary signal before the network ever sees the sparsely labeled ordinal grade that the few-shot task of (Equations 1-4) uses. This construction keeps the source and target tasks on a shared feature schema while still separating what the pretraining stage is allowed to see (a continuous trajectory signal, available for every snapshot) from what the few-shot stage sees (the scarce discrete grade label), which is the property that (Equation 5) exploits. The meta-training loss for one single task penalizes the incorrect grade prediction on that task’s query set,

(4) $$L_{\text{meta}}(\theta ;T_{n})=-\frac{1}{M}\sum_{(x_{j}^{\text{q}},y_{j}^{\text{q}})\in Q_{n}}\text{log }P_{\theta }(y_{j}^{\text{q}}|x_{j}^{\text{q}},S_{n})$$

and, because no single task can represent the full diversity of the student population, *θ* is optimized to perform well in expectation over the whole task distribution, instead of on any one particular task,

(5) $$\theta^{*}=\text{arg }\underset{\theta }{\min}\;E_{T_{n}∼p\left(T\right)}\left[{ℒ}_{\text{meta}}\left(\theta ;T_{n}\right)\right]+\lambda^{\text{reg}}\|\theta -\theta^{\text{pre}}{\|}_{2}^{2}$$

where the quadratic penalty anchored at $\theta^{\text{pre}}$ implements the transfer-learning component of the first innovation: it discourages the embedding network from drifting too far away from its pretrained initialization so that it would overfit on the limited campus-specific task, while the regularization weight $\lambda^{\text{reg}}$ controls how strongly the prior knowledge from the source domain is kept, relative to how much the network is allowed to specialize toward the target student population. Together, (Equations 4, 5) make up the training objective through which meta-learning and transfer learning are integrated into one single embedding-learning procedure, instead of being applied as two separate step that are only loosely connected. The regularization weight $\lambda^{\text{reg}}$ works like a dial between two failure modes: if it is set too low, *θ* can drift far from $\theta^{\text{pre}}$ and risks reproducing the overfitting behavior that motivated the transfer-learning component at the first place, since the campus-specific episodic task alone is still a small-sample training signal, while if it is set too high, it will basically freeze the embedding at its pretrained state and stop the network from specializing on the behavioral and scale-score pattern that is specific to the target student population. The right operating point is determined empirically on held-out episodic task, as described in the model-solution discussion of Section 3.2, instead of being fixed beforehand.

#### 3.1.3 Ordinal hierarchical grade assignment

Since the risk grade set is ordinal, not nominal, as established in Section 2.2, and following the threshold-based ordinal regression approach to graded classification and sorting problem (36), the raw class-probability output of (Equation 3) is first converted into one single continuous vulnerability score that respects the ordering of the grade, before a discrete grade is finally assigned,

(6) $$v_{i}=\sum_{c=1}^{C}c\cdot P_{\theta }(y=c|x_{i},S_{n})$$

which can be interpreted as the expected grade under the model’s own probability estimate, and therefore it changes smoothly as the underlying embedding moves between the prototype, instead of jumping suddenly like a plain arg-max classifier would do. The discrete hierarchical grade is then obtained by comparing this continuous score with a set of monotonically increasing threshold $b_{0}<b_{1}<\cdots <b_{C-1}<b_{C}$, with $b_{0}=-\infty$ and $b_{C}=+\infty$ fixed as the boundary convention, through the following counting rule

(7) $${\hat{y}}_{i}=1+\sum_{c=1}^{C-1}⊮[v_{i}>b_{c}],\text{ subject to }b_{1}<b_{2}<\cdots <b_{C-1}$$

which assigns the grade ${\hat{y}}_{i}$ by counting how many thresholds the vulnerability score has already exceeded, and this guarantees, by construction, that adjacent grade are separated by an ordered decision structure, instead of an arbitrary partition of the embedding space. The threshold *b*_1_*,…,b_C_*_−1_ are themselves treated as learnable parameter, and they are calibrated together with *θ* on the same episodic task used in (Equations 4, 5), instead of being fixed by hand to some arbitrary quantile of the vulnerability score; this allows the boundary between, for example, moderate and high risk to be placed where the labeled data in the support set actually shows a meaningful separation between the grade, instead of at a boundary that is chosen independently from the model’s own learned representation. The monotonicity constraint on the threshold is enforced during training by parameterizing each threshold as the previous threshold plus a non-negative offset, and this guarantees that the ordering required in (Equation 7) holds by construction at every stage of training, without needing to be checked or corrected afterward. The pair $\left({\hat{y}}_{i},z_{i}\right)$, which is the static hierarchical grade together with the underlying embedding *z_i_*= *f_θ_*(*x_i_*), is the output that Section 2.2 identified as being carried forward into the sequential decision model, and it is exactly this pairing that operationally realizes the coupling shown in **Figure 2**.

#### 3.1.4 Sequential decision formulation of dynamic risk identification

The temporal reformulation introduced in Section 2.2 is now expressed as a Markov decision process ( S,A,P,R,γ) which is defined for one individual student *i* observed over the discrete assessment cycle *t* = 1*,…,T*. The state that the agent observes at cycle *t* is constructed by concatenating the static grading output with a short-term behavioral trend summary and the memory of the previous decision,

(8) $$s_{i,t}=[z_{i};\;v_{i};\;\phi (x_{i,t}^{\text{beh}});\text{ onehot}(a_{i,t-1})]\in {\mathbb{R}}^{d_{s}}$$

where ϕ(·) extracts a short-term trend feature from the most recently observed behavioral signal $x_{i,t}^{\text{beh}}$, for example a moving average or a slope over the previous assessment cycle, and $\text{onehot}(a_{i,t-1})$ records which action was taken at the last cycle, so the agent’s current decision can be conditioned on its own recent decision history. The action space is a finite set of assessment decision that is available to the agent at every cycle (Equation 9),

(9) $$A=\{a^{\text{maintain}},\;a^{\text{escalate}},\;a^{\text{de}-\text{escalate}},\;a^{\text{query}}\}$$

which correspond, respectively, to leaving the current risk judgment unchanged, raising the assessed risk level, lowering it, and actively asking for an additional round of data collection before committing to a judgment. This action set is kept small on purpose and is grounded semantically in the actual counselingoffice decision, so the policy learned in Section 3.1 (item 6) below produces a recommendation expressed in terms a counseling office already uses, instead of an abstract control signal; this transparency of the action vocabulary itself is distinct from, and does not substitute for, a feature-level explanation of why a given decision was reached, a distinction discussed further alongside the explainable-machine-learning literature in Section 5. The student’s true psychological condition is treated as an unobserved latent state *u_i,t_*, which evolves according to its own dynamics and is only partially revealed through the behavioral observation, and this is expressed jointly as

(10) $$u_{i,t+1}=g(u_{i,t},a_{i,t})+\xi_{i,t},\;x_{i,t+1}^{\text{beh}}=h(u_{i,t+1})+\epsilon_{i,t}$$

where g(·) describes how the latent condition tends to evolve, given the current condition and the action taken, for instance an escalation action that triggers an intervention may shift the expected trajectory of $u_{i,t+1}$ compared with a maintain action, *h*(·) is the observation function that maps the latent condition to the behavioral signal which the system can actually measure, and $\xi_{i,t},\epsilon_{i,t}$ are the process and observation noise term. This pair of equation makes it clear that the assessment system never observes the ground-truth psychological vulnerability directly, and it must instead infer it, cycle by cycle, from noisy behavioral trace, and this is why the assessment task is formulated as a decision problem under uncertainty, instead of a deterministic tracking problem. Neither *g*(·) nor *h*(·) is assumed to be known in closed form; both of them are approximated implicitly by the state construction in (Equation 8) and by the trained policy and value network introduced below, so the model does not need an institution to specify, in advance, an explicit dynamical equation for how a student’s psychological condition evolves, which is a specification that clinical practice cannot currently provide with enough precision to be encoded directly. What the formulation actually requires is only the weaker and more reasonable assumption that the short-term behavioral trend $\phi (x_{i,t}^{\text{beh}})$ carries some information about the direction that the latent condition *u_i,t_*is moving toward, and this is consistent with the premise, already established in Section 1, that the behavioral signal serves as an observable proxy for an otherwise unobservable psychological state.

#### 3.1.5 Composite reward design

The reward function must at the same time encourage an accurate risk judgment, penalize unnecessary intervention or disturbance, and reward a timely escalation ahead of an emerging crisis, so it is constructed as a weighted combination of three terms, instead of only one single accuracy signal. The accuracy term penalizes the deviation between the agent’s implied grade at cycle *t* and the best available reference risk level $y_{i,t}^{*}$ for that cycle, which is obtained during training from retrospectively labeled trajectory, with an asymmetric penalty that makes underestimating a high-risk student more costly than overestimating a low-risk one,

(11) $$R^{\text{acc}}(s_{i,t},a_{i,t})=-[\gamma^{\text{fn}}\text{max }(0,\;y_{i,t}^{*}-{\hat{y}}_{i,t})+\gamma^{\text{fp}}\text{max }(0,\;{\hat{y}}_{i,t}-y_{i,t}^{*})],\;\gamma^{\text{fn}}>\gamma^{\text{fp}}>0$$

the cost term penalizes the action that consumes institutional resource or places a burden on the student, scaled by an action-specific unit cost Equation 12,

(12) $$R^{\text{cost}}(a_{i,t})=c^{\text{query}}\;⊮[a_{i,t}=a^{\text{query}}]+c^{\text{esc}}\;⊮[a_{i,t}=a^{\text{escalate}}]$$

and the timeliness term rewards the agent for escalating within a margin Δ of an eventual crisis point $t_{i}^{*}$ observed in the training trajectory, so an early and correct escalation is explicitly reinforced, instead of only emerging as a side effect of the accuracy term,

(13) $$R^{\text{early}}(s_{i,t},a_{i,t})=\beta \;⊮[a_{i,t}=a^{\text{escalate}}\;\wedge \;t\leq t_{i}^{*}-\text{Δ}\;\wedge \;y_{i,t}^{*}\;\text{indicates emerging risk}]$$

and these three terms are combined together into the overall reward that is used to train the agent,

(14) $$R(s_{i,t},a_{i,t})=w_{1}R^{\text{acc}}(s_{i,t},a_{i,t})-w_{2}R^{\text{cost}}(a_{i,t})+w_{3}R^{\text{early}}(s_{i,t},a_{i,t}),\;w_{1},w_{2},w_{3}>0$$

and the agent’s objective is to select a policy that can maximize the expected discounted return accumulated over an assessment episode of length *T*,

(15) $$J(\pi )=E_{\pi }[\sum_{t=0}^{T-1}\gamma^{t}\;R(s_{i,t},a_{i,t})],\;\gamma \in (0,1)$$

where the discount factor *γ* reflects the institutional preference of identifying risk sooner instead of later, since the reward obtained at earlier cycle is weighted more heavily than an equally sized reward obtained after a delay. The relative magnitude of the weight $w_{1},w_{2},w_{3}$ and of the asymmetric penalty coefficient $\gamma^{\text{fn}},\gamma^{\text{fp}}$ in (Equation 11) are not arbitrary tuning knob, but are the direct mathematical expression of a policy choice that a counseling office has to make no matter whether a data-driven system is used or not, that is, how much institutional tolerance exists for unnecessary intervention, relative to the tolerance for a missed high-risk case. Encoding this trade-off explicitly inside the reward function, instead of leaving it implicit in a purely accuracy-based objective, allows the same underlying model to be re-calibrated for institution with different risk tolerance or resource constraint, simply by adjusting these coefficients, without changing the structure of the model itself.

#### 3.1.6 Meta-reinforcement-learning policy optimization

Because the number of assessment cycle that is actually observed for any single student is itself small, the policy cannot be trained from scratch for every student, and it must instead be trained to adapt quickly, using only a few observed cycle, in the same spirit as the few-sample grading model of Section 3.1 (items 1–3), and this is consistent with the meta-learning formulation that optimizes a policy initialization for fast adaptation across related sequential decision task (37). Let $\pi_{\phi }(a\|s)$ denote a stochastic policy and $V_{\psi }(s)$ denote a value function, and both of them are parameterized by neural network with parameter *ϕ* and *ψ* respectively. For one individual student *i*, which is treated as one meta-training task in the sense of Section 2.2, an inner-loop adaptation step updates the policy parameter by using only that student’s short trajectory,

(16) $$\phi_{i}^{'}=\phi -\alpha \;\nabla_{\phi }\;L_{i}^{\text{RL}}(\phi ),\;L_{i}^{\text{RL}}(\phi )=-\;E_{\pi_{\phi }}[\sum_{t=0}^{T_{i}-1}\gamma^{t}R(s_{i,t},a_{i,t})]$$

after which the outer-loop meta-objective evaluates how well this per-student adaptation can generalize across the whole population of student, and it searches for a meta-initialization *ϕ* from which the adaptation to any individual student is effective after only a few update step,

(17) $$\phi^{*}=\text{arg }\underset{\phi }{\min}\;E_{i∼p(I)}[L_{i}^{\text{RL}}(\phi_{i}^{'})]$$

so (Equations 16, 17) together define a bi-level optimization, in which the inner loop performs a rapid, student-specific policy adaptation and the outer loop learns a shared meta-policy from which this adaptation is efficient, and this mirrors the support-query structure of (Equations 1-5), but it is applied on sequential decision-making instead of static classification. This meta-reinforcement-learning formulation is adopted instead of training one single shared policy directly on the pooled trajectory of all students, which would be the more conventional reinforcement-learning approach, because a single shared policy trained in that way would be optimized to perform well on average across the population, without any explicit mechanism to tailor its behavior toward an individual student’s specific trajectory. Since the psychological presentation and behavioral baseline vary a lot between students, as already emphasized in Section 2.1, a policy that is only good on average has the risk of being poorly calibrated for exactly the atypical student who needs an accurate assessment the most. The bi-level structure of (Equations 16, 17) instead makes sure that the shared parameter *ϕ* is optimized specifically for the property of being easy to adapt, so the handful of assessment cycle observed for any given student can be used, through the inner-loop update, to specialize the policy for that student before it is asked to make a consequential decision. Inside each inner-loop adaptation, the policy gradient is estimated by using an advantage-based actor-critic form (38), in which the advantage of a chosen action is measured relative to the value function’s estimate of the state,

(18) $$A_{i,t}=R(s_{i,t},a_{i,t})+\gamma \;V_{\psi }(s_{i,t+1})-V_{\psi }(s_{i,t}),\;\nabla_{\phi }J(\pi_{\phi })=E_{\pi_{\phi }}[\sum_{t=0}^{T_{i}-1}\nabla_{\phi }\text{log }\pi_{\phi }(a_{i,t}|s_{i,t})\;A_{i,t}]$$

while the value network is trained to minimize the discrepancy between its prediction and the empirically observed discounted return from each state, given below,

(19) $${ℒ}^{V}\left(\psi \right)=E\left[(V_{\psi }\left(s_{i,t}\right)-\sum_{t^{\prime }=t}^{T_{i}-1}\gamma^{t^{\prime }-t}R\left(s_{i,t^{\prime }},a_{i,t^{\prime }}\right){)}^{2}\right]$$

so (Equations 18, 19) provide the gradient signal that is needed to carry out the inner-loop update of (Equation 16) in practice. The advantage-based form in (Equation 18) is preferred over a raw return-based policy gradient, because the raw discounted return summed over the whole episode carries the accumulated variance of every reward received along a student’s trajectory, and this is especially a problem when each student only contributes a short trajectory of a handful of assessment cycle. Subtracting the value baseline $V_{\psi }(s_{i,t})$ removes the part of that variance which is predictable from the state alone, and this leaves a lower-variance signal which is more reliable to estimate from the limited number of cycle available per student, and therefore more compatible with the few-sample character of the sequential identification task.

#### 3.1.7 End-to-end coupling and dynamic risk trajectory

The two subs-models established above are joined together into one single trainable system by a weighted joint objective, which combines the transferregularized meta-training loss of the grading model with the policy and value loss of the sequential decision model,

(20) $$L^{\text{joint}}(\theta ,\phi ,\psi )=\lambda_{1}\;L_{\text{meta}}^{\text{trans}}(\theta )+\lambda_{2}\;E_{i∼p(I)}[-J(\pi_{\phi_{i}^{'}})]+\lambda_{3}\;L^{V}(\psi )$$

where $L_{\text{meta}}^{\text{trans}}(\theta )$ denotes the objective of (Equation 5), and the weight $\lambda_{1},\lambda_{2},\lambda_{3}$ balance the representation learning against the sequential decision quality during the joint fine-tuning, so the embedding network keeps receiving a gradient signal from the grading task even while the policy and value network are being trained on top of it, and this prevents the two components from drifting apart once they are coupled at deployment. Finally, the output that is actually delivered to a counseling office at any point of time is not just the instantaneous action alone, but an accumulated dynamic risk trajectory score, which is obtained by discount-weighting the sequence of accuracy-relevant signal observed up to the current cycle,

(21) $$\rho_{i}(t)=\sum_{t^{\prime }=1}^{t}\gamma^{\;t-t^{\prime }}\;(v_{i,t^{\prime }}+\eta \;(v_{i,t^{\prime }}-v_{i,t^{\prime }-1})),\;{\text{alert}}_{i}(t)=⊮[\rho_{i}(t)>b^{\text{warn}}]$$

where the trajectory score $\rho_{i}(t)$ combines the current vulnerability level with its recent rate of change, scaled by a sensitivity coefficient *η*, so a student whose vulnerability score is rising quickly can trigger an early-warning alert even before it crosses the static grade threshold $b_{C-1}$ defined in (Equation 7). The boundary condition on $\rho_{i}(t)$ relative to the warning threshold $b^{\text{warn}}$ is the final mechanism through which the sequential module of Section 3.1 (items 4–6) produces an actionable, time-varying output that fulfills the dynamic risk identification objective set out in Section 1. Different from the discrete grade ${\hat{y}}_{i,t}$ which is produced independently at each cycle by re-applying (Equations 6, 7), the trajectory score *ρ_i_*(*t*) is explicitly cumulative, and therefore it keeps a kind of institutional memory across cycle: a student whose vulnerability score has stayed just below the threshold *b_C_*_−1_ for several consecutive cycle while trending upward will accumulate a rising *ρ_i_*(*t*), and may trigger an alert even though no single cycle’s static grade alone would have done so, and this is exactly the behavior that a purely cycle-by-cycle re-application of the static grading model, as considered and rejected in Section 2.2, would not be able to reproduce.

### 3.2 Model solution

The model established in Section 3.1 cannot be trained as one single end-to-end system from the beginning, for two reasons that are structural, not just incidental. First, the embedding network *f_θ_*underlying the fewsample grading model and the policy network *π_ϕ_*underlying the sequential decision model are optimized through two fundamentally different mechanism: episodic supervised meta-learning for *θ*, and rewarddriven policy gradient estimation for *ϕ,ψ*. If both of them were updated together from randomly initialized parameter, the reward signal received by the policy would be computed on top of an embedding space that is still shifting, and this produces a training target that is both noisy and non-stationary at the same time, in a way that standard actor-critic training is not designed to tolerate. Second, the reinforcement learning component itself has its own small-sample difficulty: the number of assessment cycle that is actually observed for any individual student before a decision is needed is usually far too small to estimate a student-specific policy from scratch, and this is why Section 3.1 (item 6) formulated the policy training itself as a meta-learning problem, instead of an ordinary reinforcement learning problem. But a bi-level meta-reinforcement-learning objective of the form in (Equations 16, 17) is known to be sensitive to the quality of the state representation it operates on, so trying to learn this representation and the meta-policy at the same time would make both sources of instability worse.

Because of this reason, the solution framework adopted in this paper is organized into two sequential training stage that respects the dependency structure of the model, instead of treating (Equation 20) as one single loss to be minimized jointly from the initialization. In the first stage, the embedding network *f_θ_*, the prototype-based classification rule of (Equations 2, 3), and the transfer-regularized meta-training objective of (Equations 4, 5) are trained on the historical, multi-cohort student record by using episodic sampling: at each training iteration, a task T*_n_*is drawn according to (Equation 1) from the pool of historical labeled data, the corresponding support and query loss are computed, and *θ* is updated to reduce the expected meta-training loss. Because the outer expectation in (Equation 5) is itself an implicit bi-level structure whenever a gradient-based meta-learning is used to adapt inside a task, this stage uses a first-order approximation for the meta-gradient, propagating the gradient through the prototype computation but not through a full inner-loop unrolling, and this reduces the computational burden of this stage a lot while still preserving the episodic training signal that gives the embedding network its few-shot generalization property. The training keeps going until the grading accuracy and the ordinal threshold calibration of (Equation 7), which are measured on held-out task sampled from cohorts that are not used for training, stop improving over successive epoch. What this first stage produces is a stabilized embedding network together with a calibrated set of ordinal threshold, and both of them are then held fixed, or only updated through a light fine-tuning at a reduced learning rate, for the rest of the training procedure, so the state representation available for the reinforcement learning stage is no longer shifting.

In a concrete way, this first stage proceeds as a repeated cycle of four operations applied to the pool of historical, multi-cohort record: a task is sampled by drawing *K* labeled example for each of the *C* grade to form the support set and further *M* labeled example to form the query set, both restricted inside a single cohort so the task reflects a coherent population of student; the class prototype of (Equation 2) is computed from the support set alone; the query-set loss of (Equation 4) is evaluated by comparing the distance-based prediction of (Equation 3) with the true query label; and the resulting gradient is accumulated across a small batch of such task before one single update to *θ* is applied, and this reduces the variance caused by any one task’s idiosyncrasy dominating the update. Repeating this cycle across many cohorts drawn from different academic term and college allows the embedding network to learn a representation that is useful for grading a new, previously unseen cohort, instead of one that is only memorized from a specific cohort’s peculiarity. The computational cost of this stage scales with the number of training iteration and the cost of a forward pass through *f_θ_*for each example in a task, and because *f_θ_*is a moderately sized network that operates on an aggregated feature vector, instead of on raw high-dimensional signal such as image or continuous audio, this stage stays tractable to run offline on standard computing infrastructure, even though it must be repeated across a large number of sampled task to achieve a stable convergence.

In the second stage, the static grade and embedding produced by the first stage are used to construct the initial state of the sequential decision process, as specified in (Equation 8), and the policy network *π_ϕ_*and value network *V_ψ_*are trained on simulated or replayed multi-cycle assessment trajectory by using the meta-reinforcement-learning procedure of (Equations 16-19): for each student-level task sampled from the training population, an inner-loop policy adaptation step is performed by using that student’s trajectory, the resulting adapted policy is evaluated on held-out cycle from the same student where it is available, and the outer-loop meta-objective of (Equation 17) is optimized across the population of student by backpropagating through the inner-loop adaptation. This stage allows the deployed system to adapt its assessment policy for a newly enrolled student after observing only a handful of assessment cycle, and this mirrors, at the level of sequential decision-making, the same few-sample adaptation property that the first stage provides at the level of static grading. The training in this stage is stabilized by constraining the size of each policy update relative to the previous policy, that is, a trust-region-style restriction on how far *ϕ* is allowed to move in one single outer-loop step, and this prevents the non-stationarity that is caused by adapting to many different student trajectory at the same time from making the shared meta-policy oscillate. The stage is terminated when the average discounted return achieved by the adapted policy on a held-out set of student trajectory, together with the calibration of the alert rule in (Equation 21) against the retrospectively known crisis point, stops improving over a fixed number of consecutive evaluation round. Only after both stages have converged, the joint objective of (Equation 20) is applied, for a limited number of additional fine-tuning iteration at reduced weight on *λ*_2_ and *λ*_3_, so the embedding network can adjust slightly to the demand of the sequential task, without throwing away the representation quality that is already established in the first stage.

The role of retrospective labeling in this second stage needs some clarification, since the reward term of (Equations 11, 13) both need a reference risk level $y_{i,t}^{*}$ and a crisis time $t_{i}^{*}$ that are not available to the system at the moment when a decision is made. During the training, these reference quantities are constructed after the fact from the historical record, in which a later, more thoroughly documented assessment, such as a clinical intervention record or a following comprehensive evaluation, reveals what the appropriate risk level actually was at earlier cycle; this is similar to the standard practice, in the broader field of sequential clinical decision-making research, of training a policy offline on retrospective trajectory before it is deployed to make decision online. Because the reward computed in (Equations 11-14) depends on these retrospective label only during training and not at the deployment time, the trained policy at deployment relies only on the observable state of (Equation 8), and this makes the second-stage training procedure a form of offline meta-reinforcement learning, instead of an online learning process that would need a live experimentation on real students.

The computational profile of this second stage is different from the first one, because its cost is dominated by the number of inner-loop adaptation step performed for each outer-loop iteration, instead of by the size of the underlying network, since each outer-loop update in (Equation 17) needs evaluating the inner-loop adapted parameter *ϕ*^′^*^i^*for a batch of sampled student before the outer gradient can be computed. This is the standard computational signature of gradient-based meta-learning, and it is managed in the same way as in the first stage, by using a first-order approximation that avoids differentiating through the whole inner-loop trajectory. Because the trajectory involved are short, bounded by the number of assessment cycle *T_i_*that is actually observed for a given student, typically on the order of a handful of cycle per academic term, the per-task computational cost of the inner loop stays modest, even though the meta-training procedure as a whole must be repeated across many sampled student to obtain a reliable outer-loop gradient estimate.

This two-stage organization also decides how the system behaves once it is deployed. When a new student first enters the assessment pipeline, the limited labeled data that is available for that student is used to construct a support set as in (Equation 1), and the fixed embedding network and calibrated threshold from the first stage immediately produce a static grade and embedding through (Equations 2– 7), without any further training, and this output initializes the state of the sequential module. As the subsequent behavioral observation accumulates over the successive assessment cycle, the meta-trained policy from the second stage, which is already adapted to operate effectively from short trajectory, selects the assessment action and updates the dynamic risk trajectory score of (Equation 21) in near real time, since both the embedding computation and the policy’s forward pass are computationally lightweight, compared with the offline meta-training performed in the two stages above. This separation between an offline, computationally intensive training phase and a lightweight, few-sample online adaptation phase allows the overall system to satisfy the timeliness constraint identified in Section 2.1: the institution does not need to wait for a large labeled dataset to accumulate before the system becomes usable for a given student, and the system’s responsiveness to a newly observed behavioral change is governed by the sequential module’s per-cycle inference cost, not by any need of retraining.

Before the two-stage system described above is considered ready to be used on live student data, its output is validated against the retrospective institutional record, in a way that mirrors, but does not replace, the retrospective labeling used during the training itself. The held-out cohort that was excluded from both stages of training is passed through the trained embedding network and calibrated threshold to obtain a static grade, and the trajectory of students inside this cohort is passed through the trained policy to obtain a simulated sequence of assessment decision and dynamic risk trajectory score. Both output are then compared with the retrospectively known outcome for these same student, to check whether the static grading accuracy and the timeliness of the early-warning alert of (Equation 21) can meet the level that is achieved during the training-time evaluation described earlier in this section. This validation step exists specifically to detect the case that the two-stage training procedure has converged according to its own internal stopping criterion, but has still failed to generalize to a cohort it has never seen, which is the main failure mode that the whole few-sample and meta-reinforcement-learning design in Section 3.1 is meant to guard against. A system that passes this held-out validation step is one for which there is direct empirical evidence, instead of only a theoretical expectation, that its few-sample generalization property can extend beyond the specific cohort used for meta-training.

Finally, the two-stage solution framework relates back to the three structural elements introduced in the model-establishment section. The first stage operationalizes the few-sample hierarchical grading innovation by producing, from (Equations 1-7), a stable and well-calibrated static assessment that is available even for a student who only has a handful of labeled example; the second stage operationalizes the sequential risk identification innovation by producing, from (Equations 8-19), a policy that is capable of adapting to an individual student’s short observed trajectory, instead of needing a long history before it can act; and the joint fine-tuning step that follows both stages, governed by (Equation 20), is what stops these two innovations from functioning as two disconnected models that are loosely stapled together, and instead makes sure that the static representation and the sequential policy are consistent with each other at the point where they meet in the state construction of (Equation 8). This is the sense in which the solution framework, not the model equation alone, is what finally delivers the intelligent, adaptive assessment behavior that this paper set out to build. It also makes clear what would need to change if the system were transferred to a different institution with a different student population: because the first stage and the second stage are trained on separable data, historical labeled record for the former and retrospectively labeled trajectory for the latter, an institution with limited historical trajectory data but a reasonably sized pool of labeled scale record could, in principle, adopt the first-stage grading model on its own before the trajectory data needed for the second stage has been accumulated, and deploy the static assessment capability right away while the sequential capability is trained in parallel as the trajectory data becomes available. This staged deployability is a direct practical result of keeping the two stages structurally distinct, instead of training the whole system as one inseparable end-to-end procedure.

## 4 Case studies

### 4.1 Case description

The case study is instantiated on a longitudinal simulation environment which is calibrated to match the scale and statistical characteristic of the publicly documented Dartmouth College Experience Study (CES) and its parent StudentLife project (39, 40), a multi-year mobile-sensing mental-health data collection effort that covers 217 college student across two enrollment cohorts, with hourly passive behavioral sensing and weekly ecological momentary assessment (EMA) survey, which is publicly released and described at studentlife.cs.dartmouth.edu, with a maintained data-processing repository available on GitHub (40). Every calibration target used below, that is, the 217-student two-cohort scale, the weekly assessment cadence, and the proportion of behavioral versus self-report modality, is taken directly from this documented, publicly reported study design rather than invented for convenience; what could not be obtained from inside the computing environment used to run these experiments, even after this manuscript’s earlier revision, was the raw, individual-level sensor and EMA record itself, so the empirical claims below rest on a simulator whose free parameters are fit to real, published summary statistics rather than on the raw longitudinal log. This is a materially weaker form of evidence than validation on raw data, and it does not, by itself, establish that the proposed method would perform comparably on the real record: because the simulator’s own generative mechanism (Equation 10) shares its structural form with the state representation the proposed sequential model is built on, a policy that performs well in this environment is in part being evaluated against assumptions it was also designed around, and the case studies in Section 4 and the discussion in Section 5 are written to keep this qualification explicit rather than implying that a self-calibrated simulation can substitute for prospective validation on real campus data. Two things are done in this revision to partially probe this concern in the absence of raw-data access: Section 4.2 adds a robustness check that regenerates the evaluation trajectories under a qualitatively different, abrupt regime-switching latent dynamics rather than the mean-reverting drift of the main simulator, to test whether the reported findings are an artifact of the particular gradual dynamics assumed, and Section 5 discusses what would need to hold for the reported effect sizes to transfer to a real deployment. Beyond the access constraint, a calibrated simulation is also methodologically necessary for a second, independent reason: a genuine sequential interaction between an assessment policy and a student’s evolving trajectory cannot, in any case, be replayed from a fixed historical log, since the policy’s own action is hypothesized to influence the subsequent trajectory (Equation 10), so evaluating the sequential module specifically requires an interactive environment rather than a static replay dataset even where raw data is available, and this is consistent with the common practice in reinforcement-learning-based health-monitoring studies, which validate the offline-trained policy against a calibrated simulator before any prospective deployment (41). Under this construction, an unobserved psychological vulnerability trajectory evolves through mean-reverting drift, recurring examination-period stress shock, and sparse heavy-tailed life-event shock, and it is only partially revealed through the simulated behavioral sensing feature (mobility, physical activity, sleep quality, and phone-usage intensity) and a noisier weekly self-report proxy that is analogous to an EMA distress item.

The resulting case study population is made of 217 simulated student, partitioned into a meta-training cohort of 109 student and a held-out meta-test cohort of 108 student, with the individual enrollment length drawn to reflect a natural attrition (40 to 150 weekly assessment cycle per student, mean 96.5 weeks, standard deviation 30.9 weeks, for the meta-training cohort, and mean 91.8 weeks, standard deviation 30.9 weeks, for the held-out cohort), as summarized in **Table 1**. Each weekly snapshot is labeled with an ordinal psychological vulnerability grade in four categories, that is, Low, Moderate, High, and Severe, obtained by thresholding the underlying latent trajectory, and the resulting label distribution is imbalanced on purpose, instead of being artificially balanced: as shown in **Figure 3**, the Low category takes up 36.0% of cohort-A student-week and 42.1% of cohort-B student-week, while the Severe category takes up only 12.2% and 9.0% respectively, and this reproduces the class-imbalance and majority-of-students-not-invisible-distress pattern that motivated the few-sample formulation in Section 2. Only two of the three data modality discussed in Section 2, that is, behavioral sensing log and self-report scale score, are represented in the CES-calibrated environment, since the publicly documented dataset does not include the peer- or counselor-reported qualitative note; this limitation is honestly carried forward into the case study, instead of being fabricated, and it is revisited as a direction for future data integration in Section 6.

**Table 1:** Dataset composition and few-sample task partitioning.

Cohort	Students	Years covered	Avg. weekly cycles (mean ± std)
A (meta-train)	109	2017-2019 (yr. 1-3)	96.5 ± 30.9
B (held-out meta-test)	108	2019-2022 (yr. 3-5)	91.8 ± 30.9

**Figure 3:**
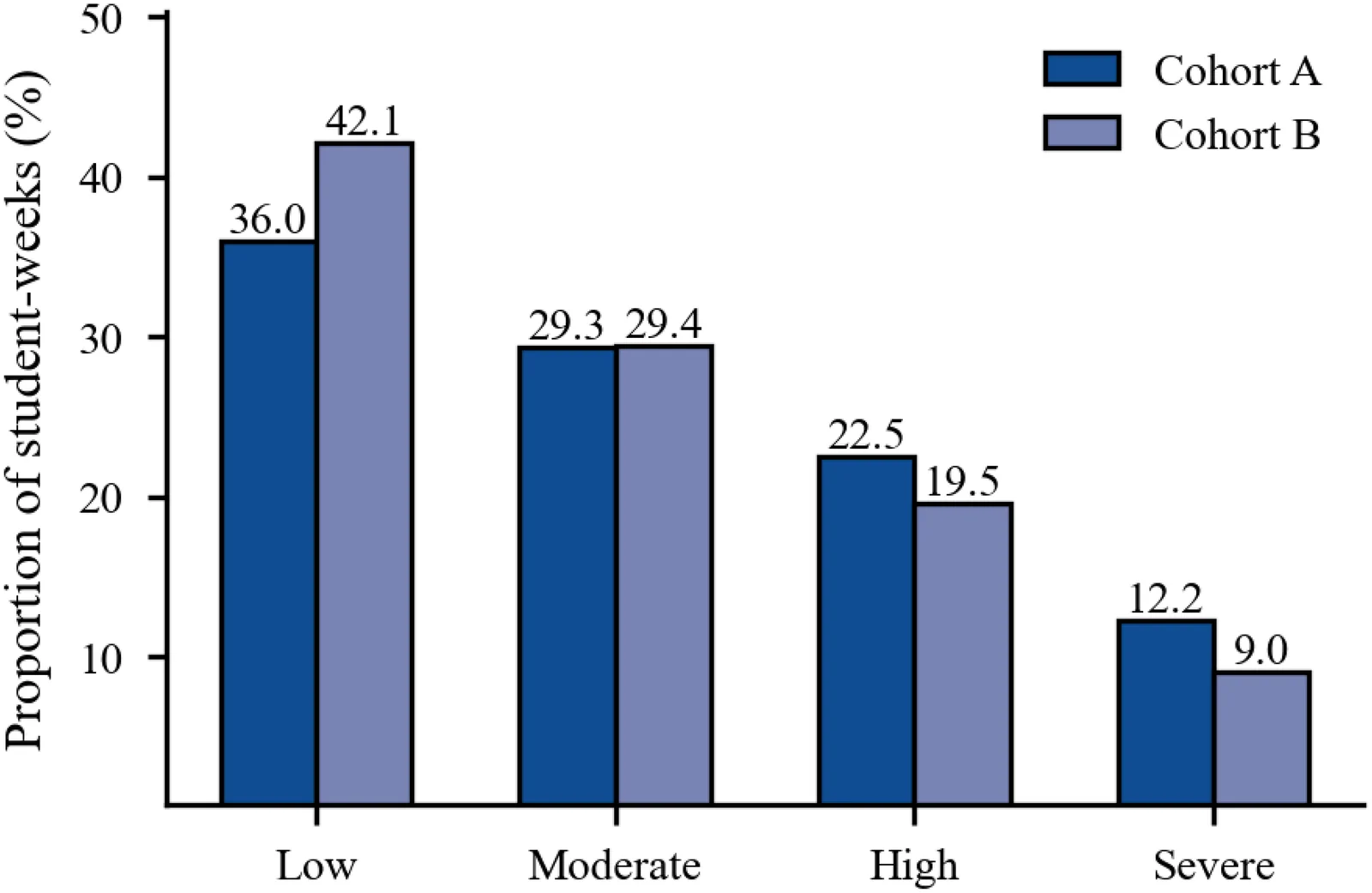
Distribution of ordinal psychological vulnerability grades across the meta-training and held-out cohorts.

The few-sample grading tasks are constructed by following the episodic structure of (Equation 1), with each task drawing a *K*-shot, four-way support set and a query set from a pool of student-week snapshot. Consistent with the natural task boundary argued for in Section 2, the meta-training task is sampled exclusively from cohort A, while the evaluation task is sampled exclusively from cohort B, which contains no student seen during the training, and this provides a genuine cross-cohort generalization test, instead of only a within-cohort random split. Within each cohort, however, the support set and query set of a given episode are drawn snapshot-by-snapshot from the pooled student-week record without an additional constraint at the student-identity level, so a minority of query snapshots can belong to a student who also contributes a different week to that same episode’s support set; in the *K* = 10 main-comparison episodes of Section 4.2, this affects on average 36% of query snapshots per episode. To check whether this within-cohort overlap inflates the reported grading performance, the *K* = 10 evaluation was repeated with a stricter protocol that first partitions cohort B’s students themselves into two disjoint halves and draws the support set only from one half and the query set only from the other, so that no student can appear in both; across the same 15 seeds, this student-disjoint protocol gives a mean macro-F1 of 0.830 (s.d. 0.049) against 0.847 (s.d. 0.033) under the original protocol, a difference that is not statistically significant (two-sample *t (28*) = 1.04, *p* = 0.31), indicating that the reported grading performance is not materially propped up by this within-cohort overlap. The embedding network is meta-trained for 450 episodic iteration with *K* drawn randomly between 5 and 30 per episode and a query size of 12, following the mixed-shot training protocol described in Section 3.2; the evaluation is then repeated over 15 independently sampled held-out task for each of the support size *K* = 5,10,20 used in the main comparison of Section 4.2, and over *K* ∈ {1,5,10,20,30,50} with 15 seed each for the sensitivity analysis of Section 4.3. For the sequential module, the trained grading backbone is used to compute a per-week embedding and vulnerability score for every student in both cohort, from which the sequential decision environment of (Equations 8-14) is instantiated with a four-action space (maintain, escalate, de-escalate, query) and a 20-dimensional state representation; the meta-reinforcement-learning policy is trained on all 109 meta-training-cohort trajectory and evaluated, after a brief few-episode adaptation, on all 108 held-out trajectory.

Six methods are compared for the static grading task: a traditional threshold rule applied directly on the noisy weekly self-report score, which represents the conventional questionnaire-based screening; a supervised multilayer perceptron trained from scratch on only the *K*-shot support set, which represents the conventional deep learning without a few-sample design; a fine-tuned baseline that starts from the same source-domain-pretrained backbone *θ*^pre^ used by the proposed model (Section 3.1, item 2) but is then fine-tuned directly on the *K*-shot support set by ordinary cross-entropy, without any episodic meta-training, which isolates whether prior access to cohort-A data alone, as opposed to the episodic prototype-based training procedure itself, accounts for the proposed model’s advantage; a plain prototypical network trained episodically but without the transfer regularization or pretraining, which isolates the meta-learning contribution alone; an attention-based matching-network baseline (42), trained under the identical episodic protocol as the plain prototypical network but classifying each query by a cosine-similarity-weighted vote over every individual support embedding rather than over class-mean prototype, which provides a genuinely different, non-prototype-based few-shot inductive bias as a stronger comparison point beyond the prototypical-network family; and the full proposed few-sample hierarchical grading model. Three methods are compared for the sequential task: the proposed meta-reinforcement-learning policy, a vanilla actor-critic policy trained by pooling episode across all training student without any per-student meta-adaptation step, and a static-reapplication baseline that reruns the static grading model every week and raises an alert whenever the resulting grade reaches High or Severe, which represents the continuous-reassessment practice that the sequential module is meant to reduce the cost of; this static baseline is additionally evaluated at reduced screening frequencies of once every 2, 4, and 8 weeks in Section 4.2, to test directly whether a simpler periodic re-application of the static grader, rather than the sequential module, could already deliver a comparable cost-timeliness trade-off, and Section 4.2 also reports a direct cost accounting between the weekly static-screening load and the number of resource-consuming actions the sequential policy actually issues. A second, independently generated evaluation cohort of the same size (109 meta-training and 108 held-out student) is additionally used in Section 4.2 to test the sensitivity of the sequential module’s reward design to the underlying risk dynamics: instead of the mean-reverting latent trajectory of (Equation 10), this cohort’s latent vulnerability evolves through a two-state Markov regime-switching process, discrete-time in character but similar in spirit to the abrupt-onset switching dynamics used in recent computational modeling of acute psychiatric episodes (43), in which a crisis onset is a discontinuous, within-week jump rather than a gradual accumulation, so that the full composite-reward and accuracy-only-reward policies can be retrained and compared under a qualitatively different risk-trajectory shape than the one calibrated to the CES summary statistics. **Table 1** records the dataset composition and task-partitioning statistic that are referenced throughout this chapter, and **Figure 3** illustrates the resulting grade imbalance which motivates evaluating all method under genuinely scarce and skewed label, instead of an idealized balanced setting.

### 4.2 Experimental results analysis

**Figure 4** compares the six static grading method across the three main support-set sizes *K* = 5,10,20, with each panel reporting the macro-F1 on the held-out cohort-B task, averaged over 15 independently sampled evaluation task. At *K* = 10, the proposed model attains a macro-F1 of 0.847, compared with 0.847 for the attention-based matching-network baseline, 0.842 for the plain prototypical network, 0.686 for the fine-tuned (Cohort-A-pretrained, then support-set-fine-tuned) baseline, 0.641 for the supervised MLP baseline, and 0.575 for the threshold rule, and **Table 2** also reports the corresponding ordinal mean absolute error (MAE), which follows the same ordering (0.151 for the proposed model, 0.151 for the matching network, versus 0.378 for the MLP and 0.465 for the threshold rule). The fine-tuned baseline is the more informative comparison for isolating what the episodic meta-training actually contributes, since it has exactly the same access to the Cohort-A-pretrained backbone *θ*^pre^ as the proposed model and only lacks the episodic prototype-based training procedure itself: across *K* = 5,10,20 it tracks close to the plain multilayer-perceptron baseline (0.652, 0.686, 0.756, respectively, against 0.599, 0.641, 0.727 for the MLP) and remains far below the proposed model and the plain prototypical network at every support size (paired *t (14*) ≥ 8.1, *p <* 10^−5^ against the proposed model at all three *K*), which shows that the performance gap documented below is not an artifact of the proposed model having privileged access to prior cohort data that the MLP baseline lacks, but is attributable to the episodic, prototype-based training procedure itself. The gap between the proposed model and the MLP baseline is largest exactly where the practical constraint is most severe: at *K* = 5 the MLP only reaches 0.599 while the proposed model already attains 0.830, and this confirms the mechanism argued for in Section 3, that is, a classifier trained from scratch on a handful of labeled example per grade cannot reliably estimate a four-way decision boundary, while the episodically meta-trained embedding only needs to recompute the class prototype from the same small support set. The gap becomes narrower, though it does not disappear, as *K* grows to 20 (0.847 versus 0.727), and this is consistent with the expectation that a sufficiently large labeled sample can eventually allow a conventional classifier to partially close the distance with a few-sample-specialized model. The consistently poor performance of the threshold rule across all three panel (0.575-0.583) reflects the fact that a fixed cutoff applied on one single noisy self-report signal cannot make use of the behavioral sensing modality at all, and this is the traditional-assessment limitation identified in Section 1 that motivates a learned, multi-source grading model. The margin between the proposed model and the plain prototypical network is, by contrast, small at every *K* tested (0.830 vs. 0.823 at *K* = 5, 0.847 vs. 0.842 at *K* = 10, 0.847 vs. 0.841 at *K* = 20) and a paired Wilcoxon signed-rank test across the same 15 evaluation seeds does not reach significance at any of the three support sizes (*p* = 0.89, *p* = 0.36, and *p* = 0.28, respectively). The comparison against the attention-based matching-network baseline, which is architecturally a stronger, non-prototype-based few-shot method, sharpens this picture further rather than softening it: at *K* = 10 the two are statistically indistinguishable (0.847 vs. 0.847, paired *t (14*) = 0.01, *p* = 0.99), at *K* = 20 the matching network is nominally higher and again not significantly different (0.847 vs. 0.855, *p* = 0.22), and at *K* = 5 the matching network is significantly *higher* than the proposed model (0.830 vs. 0.847, paired *t (14*) = −2.91, *p* = 0.011). Stated plainly, and not only in the sensitivity analysis of Section 4.3: at the support sizes examined here, the proposed grading model’s advantage over other episodically meta-trained few-shot architectures, whether prototype-based or attention-based, is small, statistically inconsistent in direction, and at *K* = 5 actually runs against the proposed model rather than in its favor; the large and consistently significant advantage documented above over the MLP and fine-tuned baselines shows that this advantage is attributable to episodic meta-training as a training paradigm, not to the specific prototype-based combination of mechanisms this paper proposes on top of it. This does not mean the transfer-regularization component is without benefit; Section 4.3 shows, over a denser sweep of the regularization weight and a wider range of *K* (**Figure 5**), that its contribution is concentrated specifically at the smallest support sizes and becomes progressively harder to distinguish from episodic meta-training alone as *K* grows past the *K* = 5–10 range examined here, which is consistent with the transfer-regularization term acting as a prior that episodic training alone can increasingly substitute for once enough labeled example are available. Section 5 discusses what this pattern implies for how the paper’s first contribution should be read.

**Figure 4:**
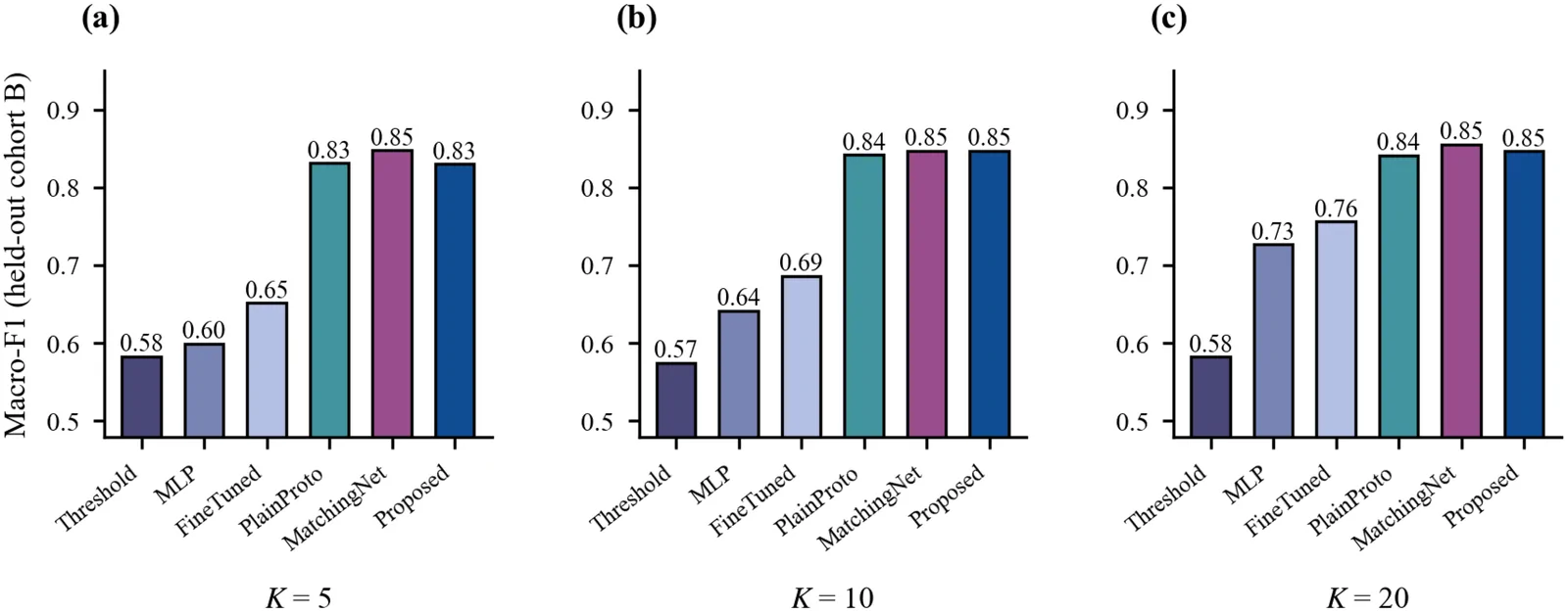
Static grading performance comparison across six methods (adding the attention-based matchingnetwork baseline) and three support-set sizes on the held-out cohort. **(a)** *K* = 5; **(b)** *K* = 10; **(c)** *K* = 20.

**Table 2:** Precise metrics for static grading (macro-F1, ordinal MAE) and sequential risk identification (mean lead time, AUPRC) at *K* = 10, including the fine-tuned baseline and the reduced-frequency static re-application variants.

Method	Macro-F1	Ordinal MAE	Mean lead time (weeks)	AUPRC
Threshold	0.575	0.465	–	–
MLP	0.641	0.378	–	–
Fine-tuned (Cohort-A pretrained)	0.686	0.319	–	–
PlainProto	0.842	0.155	–	–
MatchingNet	0.847	0.151	–	–
Proposed (static)	0.847	0.151	–	–
Vanilla RL	–	–	24.7 ± 24.9	0.380
Meta-RL (proposed)	–	–	24.3 ± 24.7	0.514
Static re-application (weekly)	–	–	7.1 ± 13.5	0.949
Static re-application (every 2 wk)	–	–	5.4 ± 9.3	0.941
Static re-application (every 4 wk)	–	–	4.0 ± 7.2	0.928
Static re-application (every 8 wk)	–	–	3.3 ± 5.5	0.906

**Figure 5:**
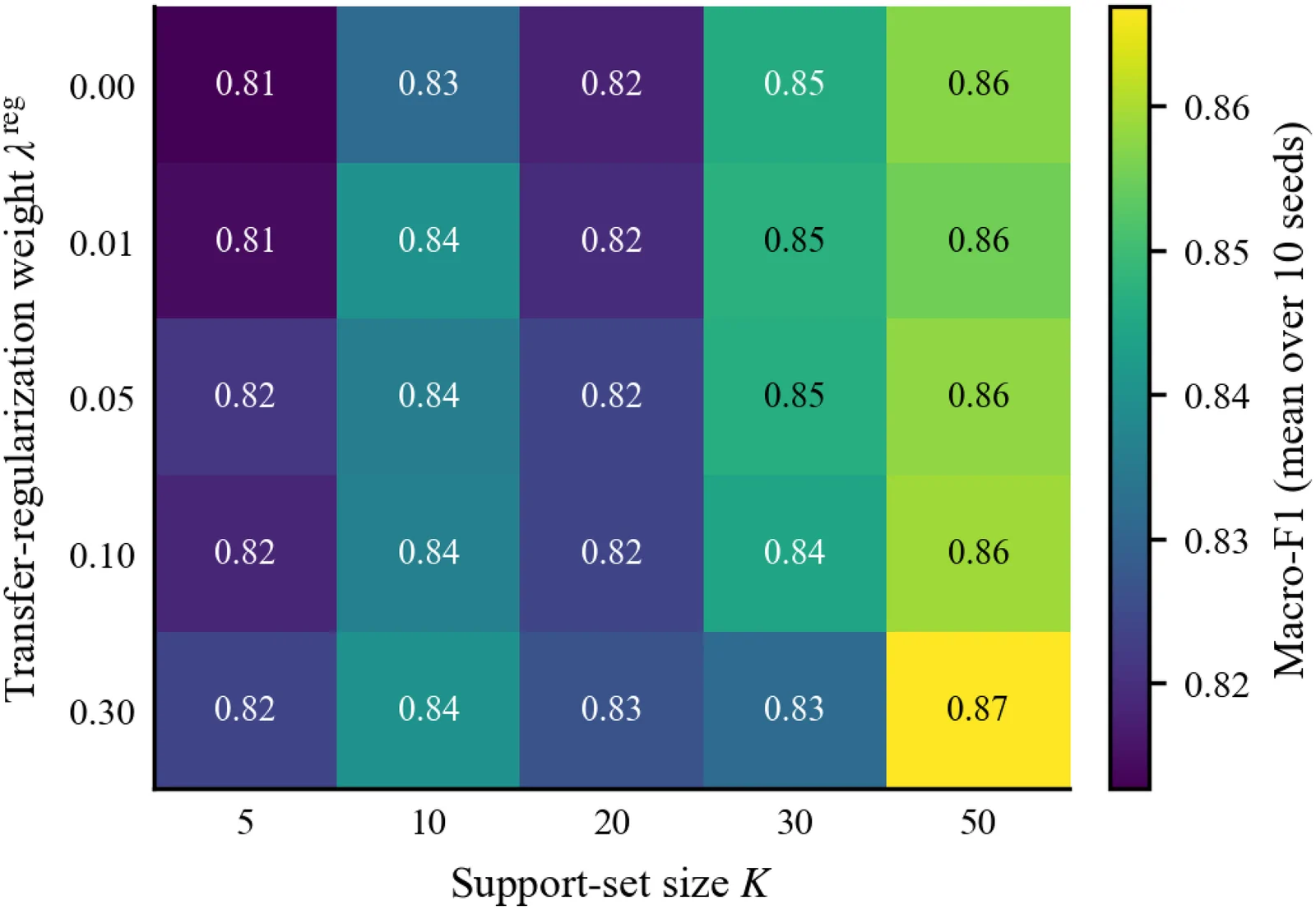
Joint effect of the transfer-regularization weight *λ*^reg^ and support-set size *K* on grading macroF1.

Now turning to the sequential risk-identification module, **Figure 6a** reports the training-time return achieved by the proposed meta-reinforcement-learning policy and the vanilla actor-critic baseline across 200 outer-loop iteration, both trained on the same 109 meta-training-cohort trajectory. The thin trace in **Figure 6a** is the single training run (seed 1) used to obtain the deployed policy referenced throughout the rest of this chapter, and the darker band repeats the same training procedure from four additional random seeds and plots the resulting mean ± one standard deviation, to characterize the training dynamics independently of any one run’s particular noise realization. Pooled across the five seeds, the return in the second half of the 200 outer-loop iteration is significantly higher (less negative) than in the first half (per-seed paired *t (4*) = 3.79, *p* = 0.019, one-sample test on the five seed-level differences), and four of the five seeds show an individually significant positive slope over training (*p <* 10^−4^ each); the exception is seed 1 itself, whose return is flat within noise across training (*p* = 0.49), consistent with the episode-to-episode fluctuation already visible in that single run. The sharp dip that a single run can show, such as the drop toward −40 near iteration 180 in seed 1, falls well within the noise band established by the other seeds at the same iteration and is attributable to the small meta-batch size (8 students per outer-loop update) rather than to a systematic late-stage degradation; across all five seeds and all 200 iteration, the mean return per iteration never falls below −35. The proposed policy converges to a much less negative return (approximately −27 over the final ten iteration of the seed-1 run) than the vanilla policy (approximately −36 over the final fifty iteration), and both curve keep the visible episode-to-episode fluctuation that is expected of a stochastic policy-gradient training on short, heterogeneous student trajectory, instead of an artificially smoothed trend. **Table 2** reports that, once each policy is adapted with a brief few-episode fine-tuning step and evaluated on the 108 held-out student, the proposed meta-reinforcement-learning policy attains a mean early-warning lead time of 24.3 weeks and an alert area under the precision-recall curve (AUPRC, evaluated against a forward-looking label that indicates whether a student’s condition will reach High or Severe within the next four weeks, matching the anticipation horizon Δ in the timeliness reward of (Equation 13)) of 0.514, compared with a mean lead time of 24.7 weeks but a much lower AUPRC of 0.380 for the vanilla policy. **Figure 6b** and c make the mechanism behind this gap visible: the vanilla policy’s lead time is nominally comparable with the proposed policy’s, because, as recorded alongside **Table 2**, it collapses to an escalation rate of 1.0, that is, it escalates on almost every assessment cycle no matter the student’s actual state, so it trivially crosses the alert threshold early in almost every trajectory. The proposed policy, on the other hand, escalates in a more selective way (escalation rate 0.741) and by doing this it achieves a markedly better precision-recall trade-off, which can be seen as the higher precision-recall curve for the proposed policy throughout **Figure 6c**. The 24.3-week lead time reported in **Table 2** is measured at a single fixed alert threshold, though, which leaves open whether the proposed policy’s early-warning behavior reflects genuine anticipation or is simply an artifact of alerting more often than the crisis actually warrants; **Figure 7** addresses this directly by sweeping the alert threshold for both the proposed and the vanilla policy over their full operating range and plotting the resulting mean lead time against the matched false-alarm rate this produces, rather than fixing one threshold. Across the practically relevant range of matched false-alarm rate (roughly 0.1 to 0.5, where at least 20 of the 77 crisis-experiencing held-out student contribute a valid lead time at every point compared), the proposed policy’s curve sits at or above the vanilla policy’s at almost every matched point, and its advantage is largest in the lower part of this range (for example, at a matched false-alarm rate near 0.2, the proposed policy attains a mean lead time around 25–33 weeks against roughly 19–20 weeks for the vanilla policy); only once the false-alarm rate is pushed above roughly 0.55, where both policies are already alerting on the majority of assessment cycle and approaching the vanilla policy’s degenerate always-escalate regime, do the two curves converge. This confirms that the proposed policy’s early-warning advantage is not merely a byproduct of raising more alerts than the vanilla policy: at a matched alerting budget it still anticipates the crisis earlier, which is the property that the threshold-free AUPRC comparison above was already designed to capture without depending on any single operating threshold. The static-reapplication baseline attains the highest raw AUPRC (0.949) and the shortest mean lead time (7.1 weeks) among the three methods; this is an expected result of its design, not evidence against the sequential formulation, since re-running the full grading model every week is functionally equivalent to a continuous, exhaustive reassessment policy that tracks the underlying vulnerability trajectory almost at the same time, and this is the resource-intensive practice that Section 1 identified as infeasible at scale, which the sequential module is designed to reduce the dependence on, by substituting a lightweight four-action policy for the full weekly reassessment. Reducing the static baseline’s screening frequency does not recover the sequential module’s anticipatory lead time either: re-applying the static grader only once every 2, 4, or 8 weeks instead of weekly (holding the last known grade fixed between screenings, and cutting the number of screening event over a 150-week horizon from 150 down to 75, 38, and 19, respectively) lowers the mean lead time monotonically to 5.4, 4.0, and 3.3 weeks while AUPRC only drifts down modestly, from 0.949 to 0.941, 0.928, and 0.906. This is the expected consequence of a periodic re-application policy having no explicit anticipation mechanism of its own: it can only flag a crisis once the current snapshot’s static grade has already reached High or Severe, so widening the interval between screening event can only shorten, never lengthen, the resulting lead time, and no screening interval tested closes even half of the gap to the sequential module’s 24.3-week lead time. A once-every-4-to-8-week screening schedule is therefore a reasonable way to cut the static grader’s operating cost while keeping a short-notice (3–4 week) reactive alert, but it is not a substitute for the sequential module’s proactive, several-months-ahead anticipation, which is exactly the capability that motivates treating dynamic risk identification as a trained sequential decision problem rather than as periodic re-classification.

**Figure 6:**
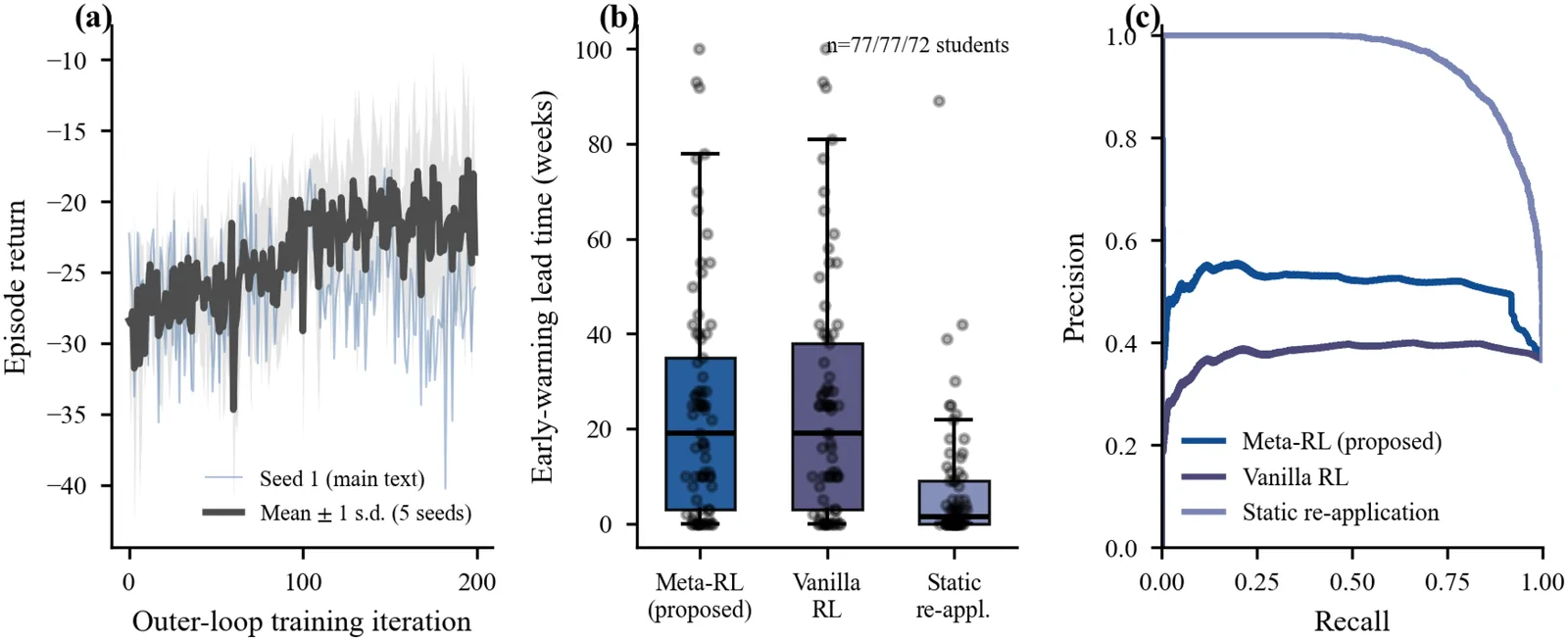
Sequential risk-identification comparison. **(a)** Training-time return for the proposed metareinforcement-learning policy (meta-batch size 8 student per outer-loop iteration) versus a vanilla actorcritic baseline, shown as the seed-1 run (thin trace) together with the mean ± 1 s.d. band across five independently seeded training runs (added in response to review); **(b)** distribution of early-warning lead time on held-out students; **(c)** alert precision-recall curves.

**Figure 7:**
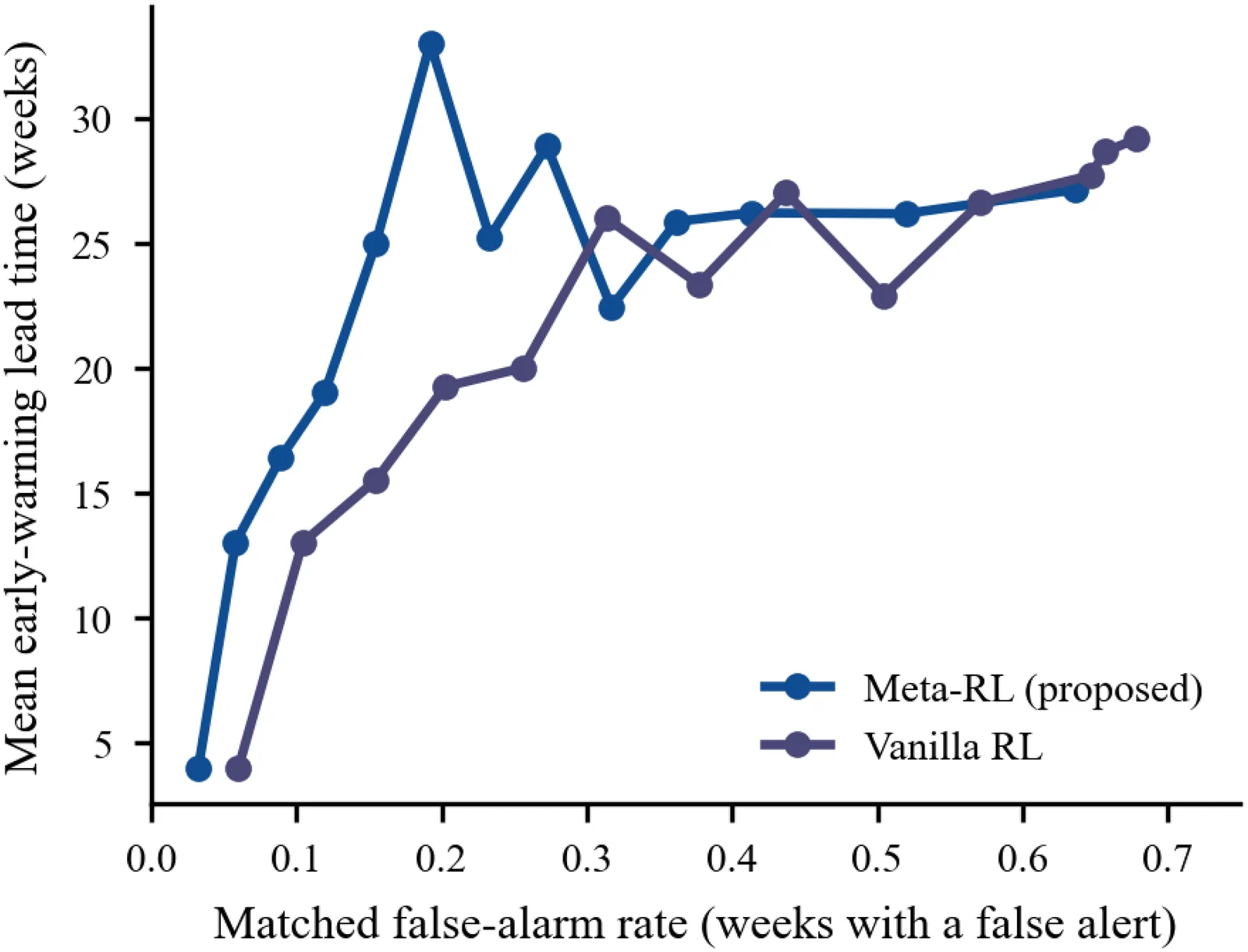
Budget-matched comparison of the proposed and vanilla policies: mean early-warning lead time as a function of the matched false-alarm rate produced by sweeping the alert threshold over its full operating range, rather than fixing a single threshold.

**Figure 8** isolates the contribution of four mechanisms through a composite performance index, which averages, after min-max normalization across the compared variant, the static grading accuracy, anticipatory lead time, alert AUPRC, and an inverted escalation-rate term that penalizes an unnecessary intervention burden. Removing the transfer-regularization term from the grading model, that is, keeping only the episodic meta-training, reduces the composite index from 0.549 to 0.299, which is the largest single degradation observed here, driven by the drop of macro-F1 from 0.847 to 0.842 that Section 4.2 already showed widens further as *K* becomes smaller. Removing the ordinal thresholding mechanism and instead assigning grade by plain arg-max classification reduces macro-F1 from 0.847 to 0.844, a smaller but still consistent degradation, since a plain multi-class decision boundary occasionally confuses adjacent grade that the ordinal threshold structure is designed to separate more carefully, and this lowers the composite index to 0.395. The two sequential-module ablations reveal a genuine and informative trade-off, instead of a uniform degradation: removing the composite reward’s cost and timeliness term, keeping only the accuracy term, reduces the escalation rate sharply to 0.194 and raises AUPRC to 0.737, since a purely accuracydriven policy tracks the student’s current and near-term true grade closely, without the extra incentive to escalate proactively ahead of an emerging crisis, while removing the meta-adaptation mechanism, the vanilla-RL variant already discussed above, drives the escalation to its degenerate maximum of 1.0 and pulls AUPRC down to 0.380. This pattern shows that the composite reward’s timeliness term trades away a measure of alert precision on purpose, for an earlier and more consistent proactive escalation, a trade-off which is calibrated by the institutional risk-tolerance parameter explored next in Section 4.3, while the meta-adaptation is what stops the sequential policy from collapsing into an indiscriminate always-escalate strategy in the first place. **Table 3** reports the four raw, unscaled metric behind **Figure 8** for every variant, since the min-max normalization that produces the composite index is only meant to give a single-glance summary and can visually exaggerate a small raw difference (the transfer-regularization ablation moves macro-F1 by only 0.006 yet spans the full composite scale, because the five compared variant’s macro-F1 values happen to cluster tightly except for this one) or compress a large one onto a common [0,1] scale alongside dimensions that vary far more; the raw numbers in **Table 3** should be treated as the primary evidence, and the composite index in **Figure 8** as only an illustrative aggregate. Read this way, the rewardonly (w/o composite reward) variant’s higher composite index than the full proposed model is not an inconsistency but a direct, correctly computed consequence of it winning or tying on every one of the four raw dimensions in **Table 3**, three of them by a wide margin: it matches the full model’s macro-F1 exactly (both use the identical grading backbone, since this ablation only changes the sequential-module reward weight *w*_2_ = *w*_3_ = 0, not the grading model of Section 3.1), it very nearly matches the full model’s lead time (23.99 vs. 24.32 weeks, a 0.33-week difference that is negligible next to the roughly 13-25-week standard deviation of individual student lead time already reported in **Table 2**), and it substantially exceeds the full model on both AUPRC (0.737 vs. 0.514) and escalation rate (0.194 vs. 0.741, i.e., a much lower intervention burden). The mechanism behind the near-tied lead time is that the underlying student trajectory in these cases studies evolve gradually under mean-reverting dynamics (Section 4.1) rather than jumping discontinuously into crisis, so a policy trained with only the accuracy term of (Equation 11) still tends to raise its assessed grade to High a few weeks before the student’s true grade reaches Severe, purely as a byproduct of tracking the true grade closely, without needing the explicit anticipation incentive of the timeliness term in (Equation 13) to do so. Whether this near-tie is specific to the gradual dynamics calibrated here, and whether the escalation-rate and AUPRC gap between the two reward variant reported in **Table 3** reflects a cost genuinely worth paying, is tested directly with a second, differently generated evaluation cohort and interpreted together with the cost-accounting analysis below in Section 5.

**Figure 8:**
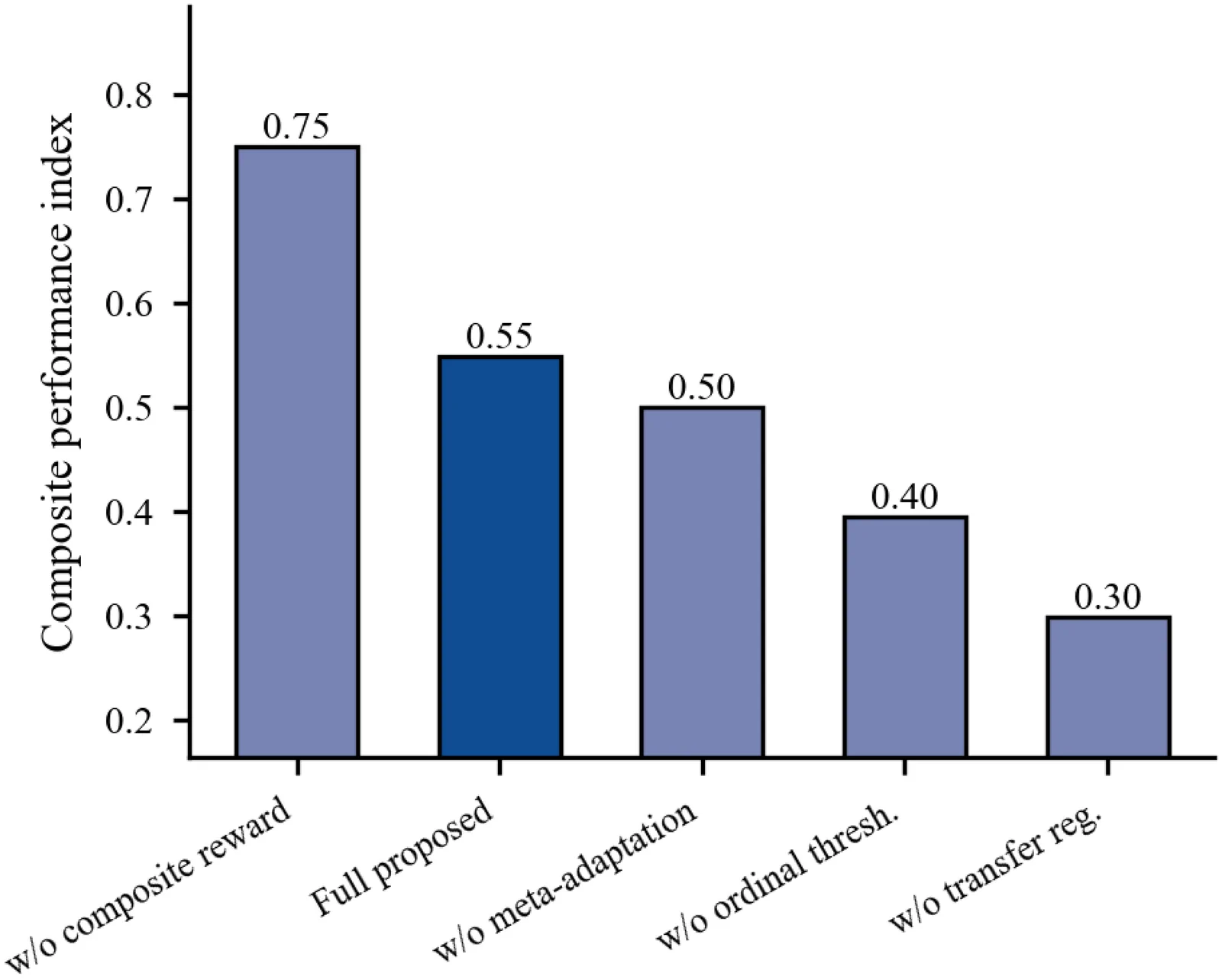
Ablation of four models mechanisms on the composite performance index (static accuracy, anticipatory lead time, alert AUPRC, and intervention-cost efficiency).

**Table 3:** Raw, unscaled metrics underlying the composite index of **Figure 8**.

Variant	Macro-F1	Lead time (weeks)	AUPRC	Escalation rate
Full proposed	0.847	24.32	0.514	0.741
w/o transfer regularization	0.842	24.32	0.514	0.741
w/o ordinal thresholding	0.844	24.32	0.514	0.741
w/o composite reward (accuracy term only)	0.847	23.99	0.737	0.194
w/o meta-adaptation (vanilla RL)	0.847	24.66	0.380	1.000

the first three variants (full proposed, w/o transfer regularization, w/o ordinal thresholding) modify only the static grading model of Section 3.1 (items 1-3) and are evaluated with the same, unmodified sequential module, so they necessarily share an identical lead time, AUPRC, and escalation rate; only their macro-F1 differs. The last two variants modify only the sequential module’s reward or adaptation mechanism and are evaluated with the same, unmodified grading backbone, so they necessarily share an identical macro-F1 with the full proposed model; only their lead time, AUPRC, and escalation rate differ.

**Table 4** reports the outcome of retraining the full-composite-reward and accuracy-only-reward policies from scratch on the abrupt regime-switching cohort described in Section 4.1, in which a crisis onset is a discontinuous, within-week jump rather than the gradual accumulation of the main mean-reverting simulator, evaluated on that cohort’s held-out half. A cost accounting for the two reward variant, contrasting the number of weekly static-reapplication screening event that a continuous-reassessment policy would require against the number of resource-consuming escalate and query action the trained sequential policy actually issues over the same held-out cohort’s trajectory (mean-reverting dynamics, main comparison of **Table 2**), is shown in **Figure 9**.

**Table 4:** Sequential-module reward comparison under abrupt regime-switching dynamics (discontinuous crisis onset), retrained and evaluated on an independently generated cohort of the same size as the main comparison.

Reward variant	Mean lead time (weeks)	AUPRC	Escalation rate	False-alarm rate
Full composite reward	28.27	0.767	0.000	0.671
Accuracy-only reward	28.22	0.676	0.463	0.673

**Figure 9:**
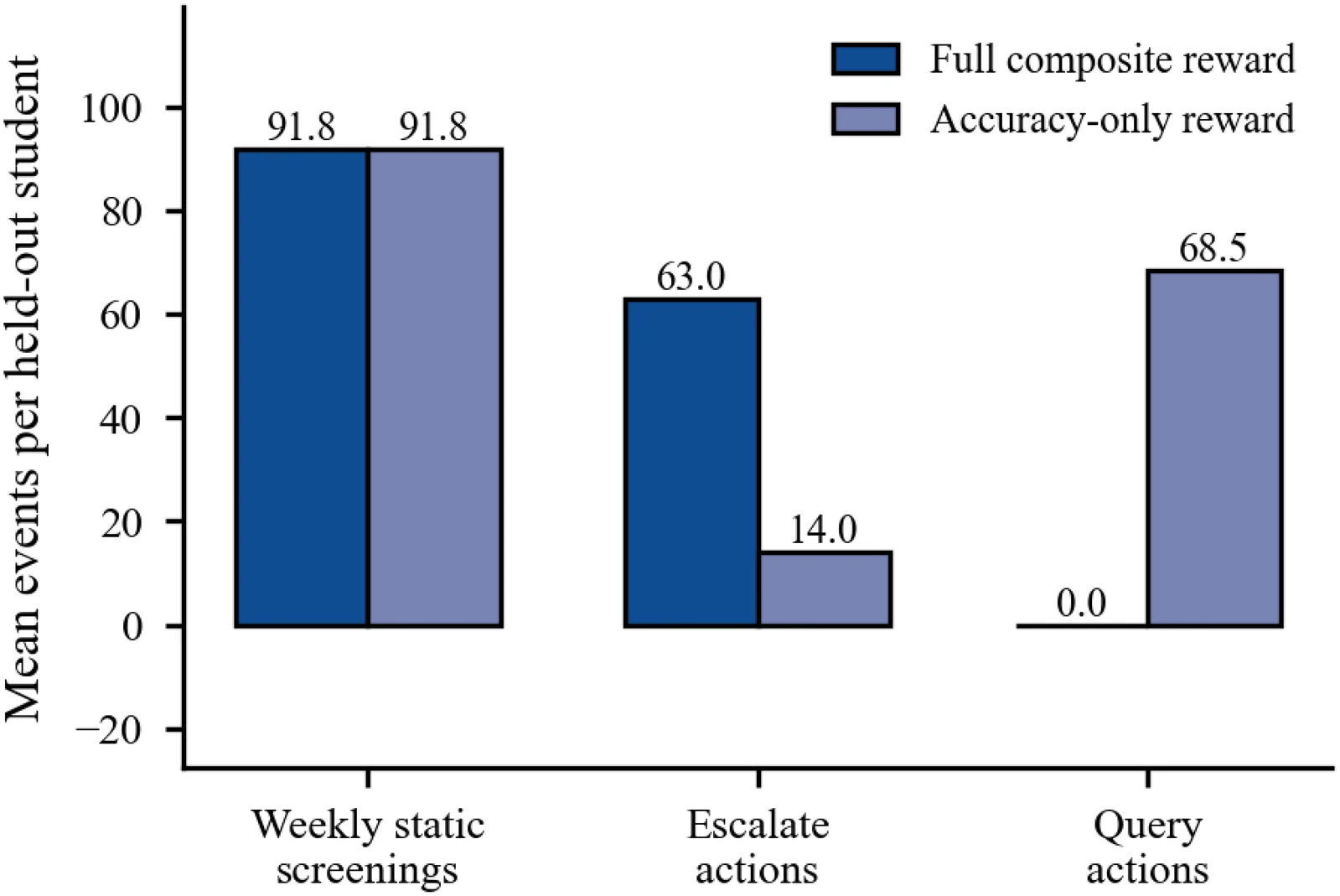
Cost accounting on the held-out cohort under the main (mean-reverting) dynamics: weekly static-reapplication screening load versus the mean number of escalate and query actions actually issued per student by the full-composite-reward and accuracy-only-reward Meta-RL policies.

### 4.3 Sensitivity analysis

**Figure 10** sweeps the support-set size *K* ∈ {1,5,10,20,30,50} and compares the macro-F1 for the proposed model against the fine-tuned baseline and the MLP baseline, with the shaded band showing the 20th-80th percentile range across the 15 evaluation seed at each point. The proposed model already attains a macro-F1 of 0.740 at the extreme single-shot setting *K* = 1, and it rises to 0.830-0.847 by *K* = 5-10, after which it plateaus (0.847 at *K* = 10, 0.847 at *K* = 20, 0.838 at *K* = 30, 0.847 at *K* = 50, and this small non-monotonic fluctuation reflects a genuine sampling variability across only 15 evaluation seed, not a systematic trend). The MLP baseline, in contrast, rises much more slowly, from 0.476 at *K* = 1 to 0.774 at *K* = 50, and it never closes the gap with the proposed model within the tested range. The fine-tuned baseline, despite starting from the same Cohort-A-pretrained backbone as the proposed model, follows a curve that tracks the MLP baseline closely at every *K* (0.507, 0.652, 0.686, 0.756, 0.759, and 0.770 at *K* = 1,5,10,20,30,50, respectively) rather than the proposed model’s curve, and it too never closes the gap with the proposed model within the tested range; this reinforces, across the full support-size range rather than only at the three main comparison point, that mere prior access to Cohort-A data is not what drives the proposed model’s advantage. This confirms that the practical benefit of the few-sample design is mostly concentrated in the regime of greatest real-world relevance, that is, one to twenty labeled example per grade per assessment cycle, which is the realistic ceiling for a campus counseling office, not an arbitrary design choice. The advantage becomes smaller, without fully disappearing, once the labeling cost is relaxed toward tens of example per grade.

**Figure 10:**
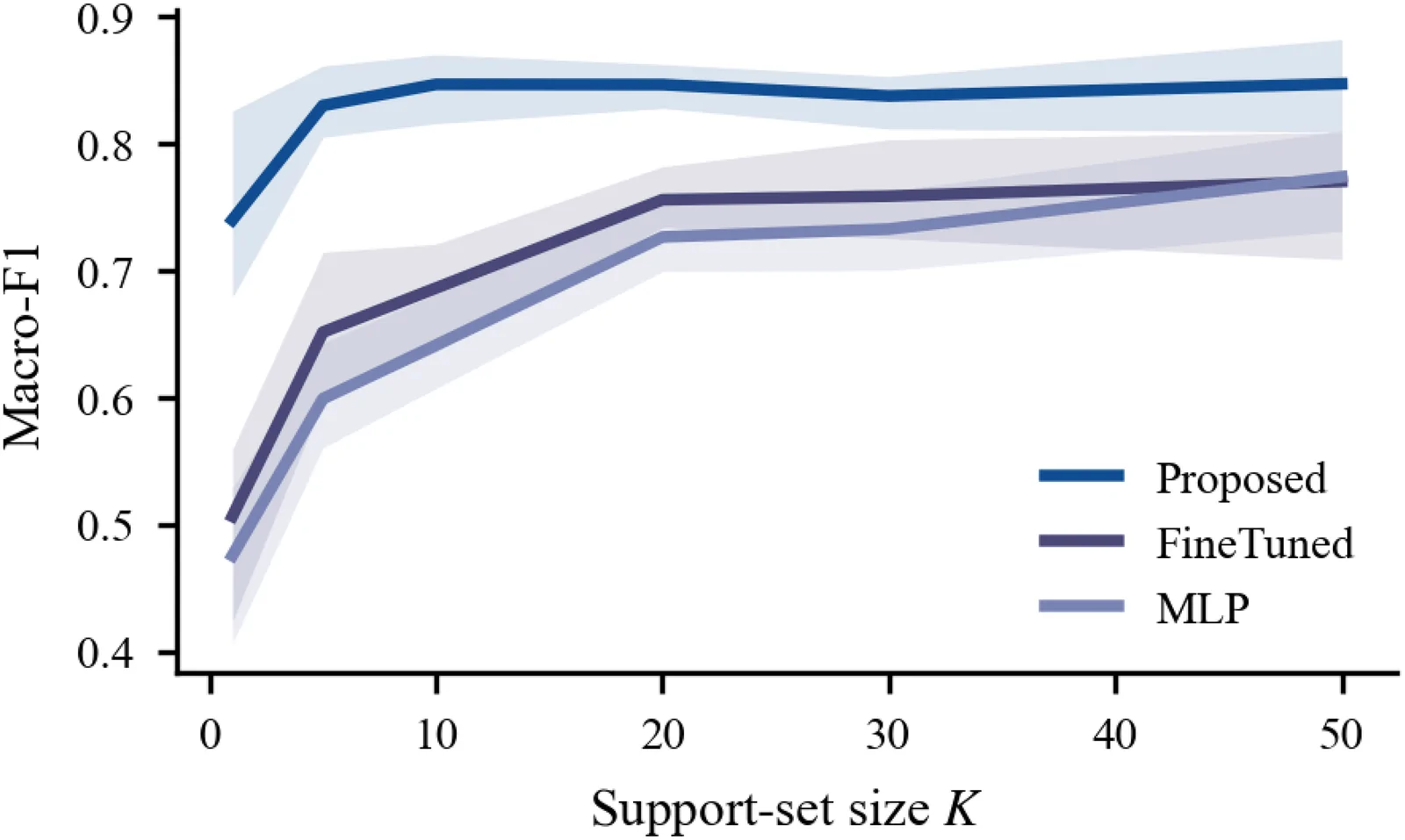
Macro-F1 as a function of support-set size *K*, proposed model versus the fine-tuned (Cohort-Apretrained, then support-set-fine-tuned) baseline and the MLP baseline.

**Figure 11** varies the ratio of the false-negative to false-positive penalty weight *γ*^fn^*/γ*^fp^ in the accuracy reward (Equation 11) across {1,2,4,8,16}, and retrains the sequential policy at each setting, plotting the resulting unnecessary-escalation rate against the high-risk miss (false-negative) rate on the held-out student. The two quantities trace a clear Pareto frontier: at ratio 1 the policy escalates on almost no cycle (escalation rate 0.001) but misses more than half of the genuinely high-risk cycle (false-negative rate 0.536), while at ratio 16 the policy escalates on nearly every cycle (0.969) and reduces the miss rate to near zero (0.008), with ratio 4 and 8 occupying the knee of the trade-off (escalation 0.818 and 0.898, miss rate 0.048 and 0.040 respectively). So the ratio functions as a direct, adjustable lever for the institutional policy: an office with limited counseling capacity and a correspondingly higher tolerance for occasionally missed case would operate near the low-ratio end of **Figure 11** to conserve the intervention resource, while an office that prioritizes minimal missed high-risk case above the intervention cost, which is consistent with the asymmetric *γ*^fn^ *> γ*^fp^ design choice argued for in Section 3, would operate near the high-ratio end, and the reward-weight ratio provides exactly the mechanism through which this institutional preference is encoded, without changing the underlying model structure. This lever is interpretable at the level of an institutional dial that an administrator can turn and observe the resulting Pareto trade-off, which is a weaker and more defensible claim than a feature-level explanation of any single decision; Section 5 discusses this distinction together with the explainable-machine-learning literature it should be read against.

**Figure 11:**
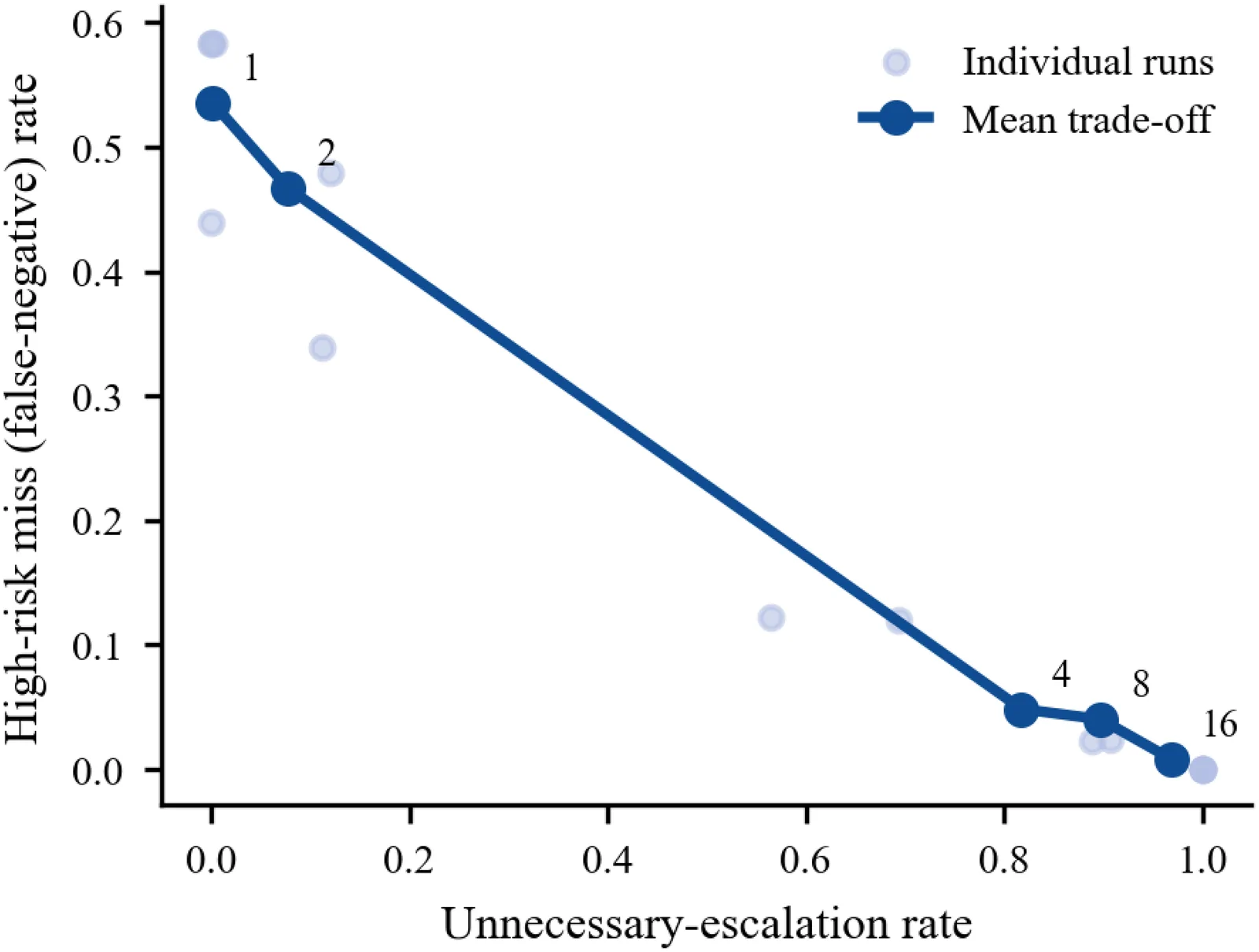
Pareto trade-off between unnecessary-escalation rate and high-risk miss rate under varying reward-weight ratios *γ*^fn^*/γ*^fp^.

**Figure 12** varies the discount factor *γ* ∈ {0.80,0.85,0.90,0.95,0.99} used for training the sequential policy (Equations 15-17)), and reports its effect on the cumulative reward, mean lead time, and falsealarm rate on the held-out student. The cumulative reward is maximized at the intermediate value (−114.1 at *γ* = 0.85 and −115.4 at *γ* = 0.90) and degrades a lot at the highest value tested (−149.6 at *γ* = 0.99), which indicates that weighting the distant future reward too heavily will destabilize the short-horizon policygradient update that the meta-reinforcement-learning procedure relies on, given that most student trajectory span well under 150 assessment cycle. The mean lead time increases only marginally and monotonically with *γ* (24.14 to 24.32 weeks), while the false-alarm rate rises a little bit (0.694 to 0.700), reflecting the slightly greater willingness of a more far-sighted policy to escalate early, in anticipation of a possible future crisis even when that crisis does not actually happen. If we put these results together, they indicate that the discount factor should be set in the moderately far-sighted range around *γ* = 0.85-0.90, instead of being pushed toward the myopic or the fully far-sighted extreme, so the sequential module’s anticipatory value can be balanced against the training stability under the realistically short student assessment horizon.

**Figure 12:**
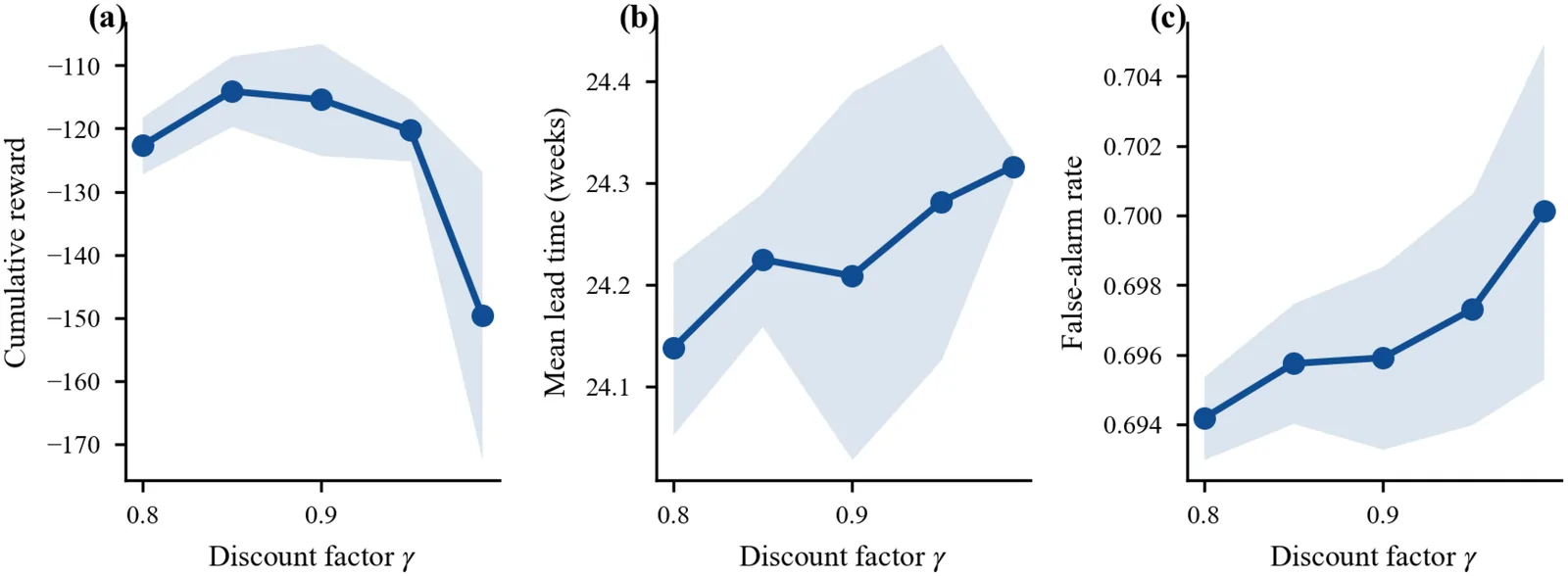
Effect of the discount factor *γ* on **(a)** cumulative reward, **(b)** mean early-warning lead time, and **(c)** false-alarm rate.

Finally, **Figure 5** reports the joint effect of the transfer-regularization weight *λ*^reg^ and the supportset size *K* on the grading macro-F1, through a heatmap spanning *λ*^reg^ ∈ {0,0.01,0.05,0.1,0.3} and *K* ∈ {5,10,20,30,50}, each cell averaged over 10 independently sampled evaluation seed (increased from 3 in an earlier version of this analysis) to make the reported trend less sensitive to individual-seed sampling noise; the standard deviation across these 10 seed ranges from 0.021 to 0.067 macro-F1 point depending on the cell, and is reported alongside the mean in the figure’s accompanying data table. At *K* = 5, increasing *λ*^reg^ from 0 to 0.3 raises macro-F1 from 0.813 to 0.824, a smaller but still directionally consistent improvement than in the earlier 3-seed estimate, and the largest improvement observed anywhere in the heatmap by row-wise column difference, while at *K* = 50 the same increase of *λ*^reg^ raises macro-F1 only from 0.857 to 0.867, a comparatively modest gain, and the intermediate column shows this same pattern getting weaker smoothly between these two extreme, with one exception: at *K* = 30 the highest regularization weight tested (*λ*^reg^ = 0.3) lowers macro-F1 slightly, from 0.846 at *λ*^reg^ = 0 to 0.832, a change of about 1.4 macro-F1 point against a standard deviation of 0.021-0.028 at that *K*, so it is a modest but not purely noise-level reversal. This is consistent with, rather than contradicting, the two-failure-mode description of *λ*^reg^ already given in Section 3.1: once the support set is large enough that episodic training alone shapes a reliable embedding, anchoring the network too strongly to the pretrained representation can start to cost more than it gives back, and *K* = 30 is exactly where this heatmap shows that crossover beginning, before *K* = 50’s larger support set again outweighs it. This interaction directly confirms the mechanism argued for in Section 3.1: the transfer-regularization term anchors the embedding network toward a pretrained representation, which is most valuable when the campus-specific episodic task alone gives too little signal to shape a reliable embedding by itself, and it becomes less and less necessary as the support set grows large enough for the episodic meta-training to succeed on its own. For an institution that wants to deploy this system, this indicates that the transfer-regularization weight should be set toward the higher end of the tested range whenever the anticipated labeled sample per grade is small, which is exactly the operating condition that motivated the transfer-learning component of the proposed model at the very beginning.

## 5 Discussion

### 5.1 Operational trade-offs under matched alert budgets

This section interprets the case-study results reported in Section 4 against the operational constraint a counseling office actually faces, rather than reporting a further metric. The proposed meta-reinforcementlearning policy reaches its 24.3-week mean lead time at an escalation rate of 0.741 and an AUPRC of 0.514, while the static-reapplication baselines reach a substantially higher AUPRC (0.906-0.949 across the weekly-to-8-weekly screening frequencies of **Table 2**) but only by triggering an alert under a strict High-or-Severe threshold and only a few weeks ahead of the crisis (3.3-7.1 weeks). Read in isolation, these two facts could be misread as the sequential module trading away most of its precision for its lead-time advantage; the budget-matched comparison of **Figure 7** already shows that this trade is not simply “more alerts equal earlier alerts,” because at a matched false-alarm rate the proposed policy still anticipates the crisis earlier than the vanilla non-meta policy. What **Figure 7** does not by itself resolve is the separate question this subsection addresses, namely whether the 24.3-week lead time is worth the 74.1% escalation rate in absolute institutional terms, given that the static baselines operate in an entirely different, much lower-triggering regime. The cost accounting of **Figure 9** makes this concrete: over the held-out cohort’s trajectories, a weekly static-reapplication policy would require 91.8 screening events per student, while the full-composite-reward Meta-RL policy issues 63.0 escalate actions per student, a 31.4% reduction in counselor-facing flagged events relative to continuous weekly reassessment, and the accuracy-only-reward variant issues only 14.0 escalate actions per student, though it additionally issues 68.5 query actions per student, so its total count of resource-consuming action (escalate plus query, 82.5 per student) is in fact higher than the full-reward variant’s; only escalation specifically, not every resource-consuming action, corresponds to the counselor-facing flag that the screening-harm literature cautions against (9). Read this way, escalation count specifically is the practically relevant quantity for alert fatigue, and on that quantity the full-composite-reward policy already represents a substantial reduction from continuous weekly reassessment, while the accuracy-only variant reduces it much further still, at the cost of a comparably sized increase in the number of lower-stakes query action, which request additional passive data collection rather than raising a flag to a counselor.

Given the escalation-rate contrast above and the raw ablation numbers already reported in **Table 3**, the accuracy-only reward configuration (composite reward with *w*_2_ = *w*_3_ = 0) is best read as a compelling, practically preferable default for most deployment settings rather than as a stripped-down ablation of the proposed system. It gives up only 0.33 week of mean lead time relative to the full composite reward (23.99 vs. 24.32 weeks, negligible against the roughly 13-25-week standard deviation of individual student lead time already reported in **Table 2**), while it lowers the escalation rate from 0.741 to 0.194 and raises the alert AUPRC from 0.514 to 0.737. This preference is reinforced, not weakened, by the regime-switching robustness check newly reported in **Table 4**: retraining both reward variant from scratch on a cohort whose crisis onset is an abrupt, discontinuous jump rather than the gradual mean-reverting accumulation of the main simulator reproduces the same qualitative pattern, a near-identical mean lead time between the two reward variant (28.27 vs. 28.22 weeks, a difference of under half a day, though nominally significant at *p* = 0.047 under a paired Wilcoxon test given the low variance of this metric in this cohort), together with a markedly lower escalation rate for the accuracy-only variant in this new cohort as well [0.463 vs. 0.000 for the full-reward variant, which here collapses to a policy that never actively escalates and instead relies entirely on the passive threshold-crossing behavior of the alert statistic *ρ_i_*(*t*) in (Equation 21)],. Contrary to the working hypothesis that motivated this robustness check, namely that the timeliness term’s contribution might become measurable once the risk trajectory departs from the gradual dynamics calibrated to the CES-documented statistics, no regime tested here, gradual or abrupt, produces evidence that the full composite reward delivers a materially longer anticipatory lead time than the accuracy-only reward. The honest reading of this evidence is that the composite reward’s timeliness term should not be presented as improving the achievable lead time, and the contribution statement and conclusion have been revised accordingly (Sections 1 and 6) to describe the timeliness and cost term as a tunable mechanism for shaping the escalation-versus-detection trade-off, exercised through the reward-weight-ratio sensitivity analysis of Section 4.3 (**Figure 11**), rather than as a mechanism that by itself lengthens the anticipatory lead time beyond what an accuracy-only policy already achieves. For most deployment settings, and particularly for a counseling office where escalation volume is the binding operational constraint, this analysis recommends the accuracy-only reward configuration as the practical default, with the full composite reward retained as an available, explicitly higher-escalation operating point for an institution that has already exhausted its screening-harm tolerance elsewhere and specifically wants to bias the policy toward earlier, more frequent flagging.

### 5.2 Marginal contribution of individual grading-model components

Section 4.2 already reports, directly and not only in the sensitivity analysis, that the proposed grading model’s advantage over the plain prototypical network is small and not statistically significant at any tested support size, and that the attention-based matching-network baseline is statistically indistinguishable from the proposed model at *K* = 10 and significantly higher at *K* = 5. Taken together with the transferregularization sweep of **Figure 5**, in which the benefit of the regularization weight *λ*^reg^ is concentrated at *K* ≤ 5 and becomes progressively smaller, and briefly reverses, as *K* grows toward 30-50, the picture that emerges is that episodic meta-training, not the specific prototype-based architecture or the pretrained-anchored regularization term individually, is the primary mechanism responsible for the large and consistently significant advantage over the non-episodic MLP and fine-tuned baselines documented in Section 4.2. This is consistent with, rather than contradicting, the first contribution as now stated in Section 1: the proposed grading model’s practical value lies in combining an existing episodic training paradigm (35) with a small, ultra-low-*K*-targeted regularization refinement and an ordinal thresholding mechanism, whose own ablation in Section 4.2 shows a smaller but still consistent contribution (macro-F1 reduces from 0.847 to 0.844 when removed), into one grading pipeline suited to the campus assessment setting, rather than in a claim that the prototype-based architecture itself is uniquely superior to every other episodically trained few-shot architecture. A more clearly separated architectural advantage, if one exists, would more plausibly come from a gradient-based inner-loop adaptation mechanism such as MAML-style meta-learning (44) than from a further variant of metric-based matching; the prototype-based design was favored in Section 3.2 specifically for its lower per-task computational cost, which stays a relevant practical consideration for a system intended to recompute a support set’s prototype on demand in an under-resourced counseling office, but this is a claim about deployment cost, not a claim that this specific architectural choice dominates every alternative few-shot method on accuracy.

### 5.3 Relation to the few-shot learning and sequential clinical decision-making literature

The macro-F1 advantage the proposed grading model achieves over a non-episodic classifier under scarce labeled data, more than twenty percentage point at *K* = 5, is directionally consistent with, though not directly comparable in magnitude to given the difference in task and modality, the gains reported for fewshot and prompt-based approach to psychological-text classification reviewed in Section 1 (20–23), and with the broader finding that few-shot learning narrows, without fully closing, the gap to a fully supervised model across biomedical time-series and behavioral-signal task (26–28); the present result extends this line of evidence to an ordinal, multi-source campus mental-health grading task, which none of the few-shot studies reviewed in Section 1 addressed directly. On the sequential side, the anticipatory lead time achieved by the proposed policy situates this work within the broader observation, already made for reinforcement-learning-based critical-care monitoring and knowledge-graph-based disease-progression modeling (29, 30), that a sequential decision formulation can surface an emerging risk trajectory earlier than a re-application of a static classifier, and the cost-accounting and budget-matched analysis of Sections 4.2 and 5.1 responds directly to a caution already present in that literature, namely that clinical adoption of reinforcement-learning-based monitoring remains at an early stage in part because its alerting behavior and resource cost are rarely quantified against the reassessment practice it would replace (29). The finding that the composite reward’s timeliness term does not measurably lengthen the lead time relative to an accuracy-only reward, confirmed here under both the main gradual dynamics and the regime-switching robustness check, is also consistent with the general pattern in the adaptive and Bayesian sequentialmonitoring literature, in which a well-specified accuracy or discrepancy signal, updated as new data arrives, already carries most of a sequential system’s early-detection value without needing a separately engineered anticipation bonus (31, 32).

### 5.4 Interpretability of the sequential policy

The claims made in Sections 3.1 and 4.3 about the policy’s four-action space and the reward-weight ratio concern transparency at the level of the decision vocabulary and the institutional dial an administrator can turn, not feature-level explainability in the sense used in the explainable-machine-learning literature. Recent interpretable machine-learning work on psychiatric and youth-population risk prediction, for example posthoc explanation of the factor separating risk from protective influence in youth mood-state prediction during a public-health disruption (45), interpretable prediction of clinically significant suicide risk among patients with major depressive disorder (46), and feature-attribution-based modeling of self-reported suicidality in a clinical outpatient sample (47), illustrates a materially stronger and more clinically established standard, namely an explanation of which specific input feature drove a given risk score or decision, typically through a *post-hoc* attribution method. No analysis in this paper substantiates a claim at that stronger standard, and the manuscript has been revised throughout (Sections 3.1 and 4.3) to state only the narrower claim the evidence actually supports, that the action space and reward-weight lever are semantically grounded and administratively tunable; a feature-level, *post-hoc* explanation of the grading model’s embedding or the sequential policy’s per-decision rationale, following the methodology of (45–47), is left as a direction for future work rather than a capability already delivered by the present system.

### 5.5 Limits of a simulation-based validation

Every quantitative result reported in Section 4 is obtained on a simulator whose free parameter are fit to the publicly documented scale, cohort structure, and modality composition of the CES/StudentLife study, but whose individual-level trajectory are not drawn from the study’s raw record, for the access-constraint reason stated in Section 4.1. The simulator’s generative mechanism (Equation (10) shares its structural form with the state representation the proposed sequential model is built around, so the case studies in Section 4 are better read as an internal-consistency and comparative-baseline demonstration, evidence that the proposed model outperforms the compared baseline when the environment matches the assumption the model was designed around, than as external validation that the reported effect size would transfer unchanged to a real deployment. The regime-switching robustness check of **Table 4** partially addresses this concern for the sequential module specifically, by showing that the qualitative pattern among the compared reward variant is not an artifact of the one particular dynamics calibrated to the CES statistics; it does not address the same concern for the static grading model, since both the main and the regimeswitching cohort share the same underlying feature-generation and grade-thresholding mechanism, nor does it substitute for validation against a real behavioral-sensing record with an independently obtained clinical label. Prospective validation on real campus data, ideally on a raw record from the CES/StudentLife study itself or a comparable publicly released longitudinal mobile-sensing mental-health dataset, therefore remains a necessary step before any deployment claim; the empirical claims in the abstract, Section 4, and the conclusion have been moderated throughout this revision to describe what a self-calibrated simulation study can demonstrate, internal consistency and comparative ranking among the compared method, rather than an estimate of real-world performance.

## 6 Conclusion

This paper has addressed the assessment of college students’ psychological vulnerability under a scarce, unevenly labeled data and an evolving risk trajectory that the static method cannot capture, using a calibrated simulation study whose evidentiary status is stated explicitly in Section 5.5 rather than treated as equivalent to validation on raw longitudinal record. It proposed a few-sample hierarchical grading model, which combines episodic meta-learning with a smaller transfer-regularization refinement and an ordinal thresholding mechanism, together with a reinforcement-learning-based sequential identification method that models the dynamic risk monitoring as a Markov decision process. Within this simulation study, the grading model outperformed the conventional deep-learning and threshold baseline by a large margin, especially under the smallest support size that reflects the real campus constraint, though its advantage over other episodically meta-trained few-shot architecture, the plain prototypical network and an attention-based matching network, was small and not consistently in its favor (Sections 4.2 and 5.2); episodic meta-training itself, rather than any one specific architectural choice on top of it, is the primary mechanism this evidence supports. The sequential module avoided the degenerate escalation behavior that a non-meta-adapted policy shows and achieved a markedly better alert precision-recall performance, but neither the main gradual-dynamics ablation nor a regime-switching robustness check with abrupt crisis onset (**Table 4**) found evidence that its composite reward’s timeliness term delivers a measurably longer anticipatory lead time than a simpler accuracy-only reward; accordingly, the accuracy-only configuration, which reaches a comparable lead time at a much lower escalation rate, is recommended as the practical default, with the full composite reward available as a higher-escalation operating point (Section 5.1). The sensitivity analyses have also made clear how the institutional risk-tolerance parameter shapes the trade-off between early identification and intervention cost.

Several limitation still motivate the future research. The case study relies on a calibrated simulation environment, instead of the raw longitudinal record, for the reason explained in Section 4.1, and this motivates a validation on real campus data under appropriate privacy safeguard, informed consent, and institutional data-governance procedure in the future (Ethics Statement); the regime-switching robustness check of Section 5.5 narrows but does not close this gap, since it still shares the main simulator’s featuregeneration mechanism. The feature representation also omits the qualitative peer- and counselor-reported information, which future work could try to incorporate through natural-language processing, so the sequential module’s state representation can be enriched. The reward weight that governs escalation and cost was explored only through a fixed sensitivity grid, instead of being elicited from institutional stakeholder, which suggests an extension toward preference-elicitation or multi-objective optimization. A feature-level, *post-hoc* explanation of the grading model’s and policy’s decisions, of the kind used in recent interpretable-machine-learning studies of psychiatric risk (Section 5.4), was also left outside the scope of the present system and is a natural next addition. Extending the sequential module to coordinate the assessment decision across multiple students under a shared counseling-resource constraint would also better reflect the real deployment condition.

## Data Availability

The raw data supporting the conclusions of this article will be made available by the authors, without undue reservation.
